# Global emergence of Acinetobacter baumannii International Clone 12 predominantly found in the Middle East

**DOI:** 10.1099/mgen.0.001572

**Published:** 2025-11-28

**Authors:** Nabil Karah, Nathan Faille, Nancy Allard, Frédéric Grenier, Antoine Abou-Fayad, Paul G. Higgins, Leena Al-Hassan, Benjamin A. Evans, Laurent Poirel, Rémy A. Bonnin, Anette M. Hammerum, Frank Hansen, Rayane Rafei, Monzer Hamze, Xavier Didelot, Santiago Castillo-Ramírez, Simon Lévesque, Sébastien Rodrigue, Bernt Eric Uhlin, Louis-Patrick Haraoui

**Affiliations:** 1 Department of Molecular Biology, Umeå University, Umeå, Sweden; 2 Department of Microbiology and Infectious Diseases, Université de Sherbrooke, Sherbrooke, Québec, Canada; 3 Centre de recherche Charles-Le Moyne, CISSS Montérégie-Centre, Greenfield Park, Québec, Canada; 4 Department of Biology, Université de Sherbrooke, Sherbrooke, Québec, Canada; 5 Department of Experimental Pathology, Immunology and Microbiology, American University of Beirut, Beirut, Lebanon; 6 German Centre for Infection Research (DZIF), Partner Site Cologne-Bonn, Cologne, Germany; 7 Institute for Medical Microbiology, Immunology and Hygiene, Faculty of Medicine and University Hospital Cologne, University of Cologne, Cologne, Germany; 8 Department of Global Health and Infection, Brighton and Sussex Medical School, Brighton, UK; 9 Norwich Medical School, University of East Anglia, Norwich, UK; 10 Department of Medicine, Université de Fribourg, Fribourg, Switzerland; 11 Service de Bactériologie, Hôpital de Bicêtre, Faculté de médecine Université Paris-Sud, Le Kremlin-Bicêtre, France; 12 Bacteria, Parasites and Fungi, Statens Serum Institut, Copenhagen, Denmark; 13 Laboratoire Microbiologie Santé et Environnement (LMSE), Doctoral School for Science & Technology, Faculty of Public Health, Lebanese University, Tripoli 1300, Lebanon; 14 School of Life Sciences and Department of Statistics, University of Warwick, Coventry, UK; 15 Programa de Genómica Evolutiva, Centro de Ciencias Genómicas, Universidad Nacional Autónoma de México, Cuernavaca, Mexico; 16 CIUSSS de l’Estrie-CHUS, Sherbrooke, Québec, Canada; 17 Humans & the Microbiome Program, Canadian Institute for Advanced Research, Toronto, Ontario, Canada

**Keywords:** *Acinetobacter baumannii*, carbapenem-resistant, genomic sequencing, International Clone 12, Middle East, ST158, *tet*(X3)

## Abstract

Infections caused by carbapenem-resistant *Acinetobacter baumannii* (CRAB) are of great concern, as mortality is high, and treatment options are very limited. Despite having among the highest rates reported worldwide, scarce genomic data are available on CRAB strains from the Middle East. Here, we report the global emergence of a novel International Clone (IC), designated IC12, based on the epidemiological, phenotypic and genome sequencing data (short reads and long reads) of a set of 60 *A*. *baumannii* isolates belonging to multilocus sequence type 158 (Pasteur scheme). IC12, prevailing in the Middle East since 2007, has also been found in Europe, Asia and South America. Alleles OXA-65 and ADC-117, coded by the *bla*_OXA-51-like_ and *bla*_ADC_ *A. baumannii*-intrinsic genes, respectively, were hallmarks shared by all the isolates. Plasmid pIC12-2 (80,000 bp), which carries a *repAci6* replication initiator (RP-T1) and a type IV conjugative transfer system, played a major role in the antimicrobial resistance profile of 54/60 of the IC12 isolates. This resistance was mediated by three mobile genetic elements, namely Tn*2008*, MITE*Ab-IC12* and Tn*aphA6*. All four Peruvian IC12 isolates lacked pIC12-2 and carried a different set of plasmids. Two of the Peruvian isolates carried a chromosomal resistance island of 79,396 bp long (designated IC12-RI) marked by the occurrence of *tet*(X3). The global spread of IC12 is worrying and calls for further studies on the virulence features and clinical impact of this clone.

**Impact Statement**

*Acinetobacter baumannii* is a common human pathogen with increasingly limited therapeutic options due to growing antibiotic resistance rates. Certain *A. baumannii* clones are more successful than others and are recognized as International Clones (IC) when there is evidence of global spread. In this study, we identify a novel IC of *A. baumannii*, named International Clone 12, based on extensive genomic analyses of strains belonging to sequence type 158 of the Pasteur multilocus sequence typing scheme. These strains were predominantly isolated in the Middle East, with a subset identified in Europe and Peru. In addition, we present extensive analyses of the mobile genetic elements and antibiotic resistance genes found in 60 of these strains, as well as phylogenetic reconstruction, contributing to a more detailed understanding of the evolution and spread of this clone.

## Data Summary

**Supplementary Table S1**. List of Sequence Read Archives (SRA) and BioSamples related to the BioProject number PRJNA1015678.

**Supplementary Table S2**. The geographic distribution of 110 ST158^Pas^ isolates retrieved from the literature, from co-authors’ strain collections, and/or from the PubMLST and GenBank records.

**Supplementary Table S3**. Description of 60 isolates belonging to International Clone 12, including associated epidemiological information.

**Supplementary Table S4**. Genomic characterization of 60 International Clone 12 isolates with focus on mobile genetic elements and antibiotic resistance genes.

**Supplementary Figure S1**. Nucleotide sequence alignment of the *rpoD* alleles 26 (sequence type 499) and 141 (sequence type 1717) according to the Oxford scheme for multilocus sequence typing of *Acinetobacter baumannii*.

**Supplementary Figure S2**. Amino acid sequence alignment of OXA-65 (encoded in the genomes of all our isolates except for Ab-IC12-Peru-2) and OXA-65-like (encoded in the genome of Ab-IC12-Peru-2).

**Supplementary Figure S3**. Graphic representation of MITE*Ab-IC12* and MITE*Ab-IC12-extended* as present in Ab-IC12-Kuwait-1 (A) and Ab-IC12-Belgium-1 (B).

**Supplement Figure S4**. Nucleotide and amino acid sequence alignments of the *bla*_GES-12_, *bla*_GES-22_ and *bla*_GES-35_ in reference to the *bla*_GES-11_ allele detected in the genomes of IC12s.

**Supplementary Figure S5**. Nucleotide sequence alignment of the *tet*(X3) allele that was detected in the genomes of three isolates from International Clone 12 (represented by Ab-IC12-Peru-4) and compared to the first described *tet*(X3) allele in p34AB (GenBank: MK134375.1).

**Supplementary Figure S6**. Nucleotide sequence alignment of two different *aadB* alleles detected in the genomes of five isolates (represented by *aadB*_Ab-IC12-Lebanon-1_) and six isolates (represented by *aadB*_Ab-IC12-Egypt-1_).

**Supplementary Figure S7, S8, S9, S10, S11, S12, S13, S14 and S15**. Graphic representations of pIC12-1 (a, b, c, d, e, f and g), pIC12-2 (a, b, c, d, e, f and g), pIC12-3, pIC12-4, pIC12-5, pIC12-6, pIC12-7, pIC12-8 and pIC12-9, respectively.

## Introduction

*Acinetobacter baumannii* is a Gram-negative bacterium that, over the last four decades, has become one of the most frequently encountered and problematic human opportunistic pathogens, largely due to its antimicrobial resistance profile [1]. As a cause of hospital-acquired infections, *A. baumannii* most commonly leads to ventilator-associated pneumonia, wound infections and catheter-related bloodstream or urinary tract infections. Infections due to *A. baumannii* are primarily identified in hospitalized patients, with a propensity for those who are immunocompromised or in intensive care units [2]. It is also reported in trauma victims with secondary infections, notably among civilians and combatants in the context of armed conflicts, including recent data from Iraq and Ukraine that highlight its emergence as a key pathogen in war-related injuries [3,4].

Carbapenem antibiotics, a class of drugs within the large family of beta-lactam antibiotics, have broad-spectrum antibacterial activity. Although the carbapenems imipenem and meropenem exhibited good activity against *A. baumannii* isolated from human infections, nowadays a growing number of *A. baumannii* encountered in clinical practice are carbapenem-resistant * A. baumannii* (CRAB) [5]. Carbapenem resistance in *A. baumannii* often occurs by acquisition of an accessory Ambler class D gene, such as *bla*_OXA-23-like_, *bla*_OXA-24/40-like_, *bla*_OXA-58-like_, *bla*_OXA-143-like_ and/or *bla*_OXA-235-like_, or through upregulation of the intrinsic *bla*_OXA-51-like_ gene [6,8]. In addition, several Ambler class A and B carbapenemases have been reported in *A. baumannii*, including *bla*_NDM_, which most likely originated in an *Acinetobacter* background [9,11], and particular alleles of *bla*_GES_ [12]. Due to the widespread circulation of mobile genetic elements carrying carbapenemase genes among *A. baumannii*, and the subsequent clonal expansion of carbapenem-resistant strains, some regions of the world have CRAB rates exceeding 70% [13]. This worrisome trend has led the World Health Organization to list CRAB as one of its three critical priority pathogens for research and development of new antibiotics [14,15].

In *Acinetobacter*, some strains associated with hospital outbreaks are widely distributed across multiple countries and are referred to as International Clones (IC). Each IC can be categorized based on multilocus sequence typing (MLST), using either the Pasteur (Pas) or Oxford (Oxf) scheme. Both schemes sequence seven distinct housekeeping genes, allowing for precise identification and tracking of these clones. The global clinical population of *A. baumannii* is currently dominated by a few epidemic clones, especially IC2, also known as global clone 2 [16,17]. IC2 corresponds to clonal complex (CC) 2^Pas^ in the Pasteur scheme, as well as CC208^Oxf^, CC218^Oxf^ and CC281^Oxf^ in the Oxford scheme [18]. Other worldwide prevalent clones include IC1 (CC1^Pas^ and CC231^Oxf^), IC6 (corresponding to sequence type (ST) 78^Pas^ and ST944^Oxf^), IC7 (CC25^Pas^ and CC229^Oxf^) and IC8 (CC10^Pas^ and CC477^Oxf^) (https://acinetobacterbaumannii.no/overview/global-epidemiology/ [19,20]). IC4 (ST15^Pas^ and ST438^Oxf^) and IC5 (ST79^Pas^ and ST205^Oxf^) have also been recognized as major global epidemic lineages, especially in Latin America [21]. IC3 (ST3^Pas^ and CC928^Oxf^) is rarely encountered nowadays, while new clones, IC9 (ST85^Pas^ and ST1089^Oxf^), IC10 (CC33^Pas^) and IC11 (ST164^Pas^), have recently been reported [18,22, 23].

In this study, we present a comprehensive overview of the geographic distribution, epidemiological information and genomic data of a novel IC of *A. baumannii* represented by ST158^Pas^, including the new complete genomes of 60 representative isolates (Supplementary Material 1, Table S1, available in the online Supplementary Material).

## Methods

### Bacterial isolates

We identified a total of 110 ST158^Pas^ isolates in the literature by querying the PubMed® database for biomedical literature (https://pubmed.ncbi.nlm.nih.gov/) [12,18, 22,32], from co-authors’ strain collections and/or from the PubMLST (https://pubmlst.org/organisms/acinetobacter-baumannii) and GenBank records (Table S2). A subset of 60 isolates of *A. baumannii* belonging to ST158^Pas^ was included in this study, based on the availability of the strain or whole-genome sequencing data. The isolates were collected between 2007 and 2022 in Kuwait (*n*=28), Egypt (*n*=7), Lebanon (*n*=5), Pakistan and Peru (*n*=4 each), Jordan and Denmark (*n*=3 each), Saudi Arabia (*n*=2) and Iraq, Afghanistan, Belgium and Ireland (*n*=1 each). Four of the Lebanese isolates were cultured from Iraqi (*n*=3) or Syrian (*n*=1) citizens seeking medical care in Lebanon. The three isolates from Denmark had travel histories to Egypt (*n*=2) and Turkey (*n*=1). All the isolates, except those from Jordan, Afghanistan and some strains from Peru, were cultured from human clinical or screening specimens, including blood, respiratory tract, urine, wound and stool samples (Table S3). Samples were obtained not for this study or research but rather for diagnostic purposes.

### Whole-genome sequencing

All 60 isolates underwent short-read sequencing, of which 46 were completed by Université de Sherbrooke (UdS) (this study), 3 by co-authors [33,35] and 11 by previous studies [23,27, 36,43]. For all 46 in-house sequenced isolates, DNA isolation, library construction and genome sequencing were performed according to the manufacturer’s instructions. Following overnight growth at 37 °C in Miller’s lysogeny broth, genomic DNA was extracted using the Quick-DNA Magbead Plus Kit (Zymo Research), and DNA libraries were prepared using the NEBNext Ultra II FS DNA Library Prep Kit (New England Biolabs, NEB). DNA purification and size selection were made using Ampure XP beads (Beckman Coulter) and quantified using Quant-iT PicoGreen dsDNA Assay (Thermo Fisher). The quality and size distribution of the DNA were assessed on a Fragment Analyzer using the HS NGS Fragment Kit (Agilent). The pooled samples were then sequenced on a NovaSeq 6000 sequencer (Illumina) with paired-end 250 bp read length.

A subset of 17 isolates was selected for long-read sequencing based on the results of their strain typing, genetic resistance patterns and geographical and chronological distributions (Tables S3 and S4). All the long-read sequences were completed at UdS. Extracted genomic DNA was first treated with the NEBNext Ultra II End Repair/dA-Tailing Module (NEB). Then, barcodes from the Native Barcoding Expansion 1–12 and 13–24 from Oxford Nanopore Technologies (ONT) were ligated using the NEBNext Ultra II Ligation Module (NEB). DNA purification was made using Ampure XP beads (Beckman Coulter). The DNA from different barcoded samples was pooled, and the adapter AMII (ONT) was ligated using the NEBNext Ultra II Ligation Module (NEB). Sequencing was done with an R10.4 MinION Flow Cell using a MinION Mk1B (ONT).

### Data analysis and bioinformatics

Quality control and trimming of the Illumina reads were done using fastp 0.21.0 with –cut_right –cut_window_size 4 –cut_mean_quality 20 –length_required 30 –detect_adapter_for_pe [44]. Samples with less than 20× coverage were re-sequenced. Unicycler 0.4.9 was used to make assemblies of the trimmed Illumina short reads and ONT long reads when available [45]. Contigs were filtered to retain only those above 500 bp.

Taxonomic identification was made on the assemblies using Kraken 2 (2.0.9-beta) [46]. Two distinct MLST schemes exist for * A. baumannii*, known as the Oxford and Pasteur schemes [47,48]. The ST of each strain was determined using mlst 2.11 (Seemann T, mlst Github https://github.com/tseemann/mlst), which made use of the PubMLST website (https://pubmlst.org/) [49]. The isolates were also typed using two single-locus sequence typing schemes based on the allelic identity of their *A. baumannii*-intrinsic *ampC* and *bla*_OXA-51-like_ genes [26,50]. To assess the genetic distance between our isolates and all the other 11 known ICs, we first generated allelic profiles using chewBBACA AlleleCall 2.8.5 [51] and the *A. baumannii* core genome multilocus sequence typing (cgMLST) schema from https://www.cgmlst.org. A minimum spanning tree based on the allelic profiles was rendered with GrapeTree 2.2 using the MSTreeV2 method [52].

Antibiotic resistance genes were detected and annotated using ResFinder 4.1 [53]. *bla*_OXA_ and *bla*_GES_ variants were curated using the beta-lactamase database [54]. The presence of neighbouring insertion sequence (IS) elements was detected using ISfinder [55]. Plasmid replicase genes were detected and annotated according to the *A. baumannii* PCR-based replicon typing scheme and other relevant studies [56,58]. Long-read sequencing data were used to determine the full-length sequence of large plasmids and chromosomal resistance elements. The features were annotated manually based on similarities to the GenBank records. Search for similarities was performed using the basic local alignment search tool (http://blast.ncbi.nlm.nih.gov/Blast.cgi) [59]. SnapGene version 5.3.2 (https://www.snapgene.com/) was used to create, visualize and compare circular maps of the detected plasmids.

Various bioinformatic tools were used to assess the relationships among the isolates and to determine the evolutionary rate of ST158^Pas^. In the initial step, Snippy 4.6.0 was employed to pinpoint the variants as compared to the reference strain Ab-IC12-Kuwait-1, utilizing the assemblies as input (https://github.com/tseemann/snippy). Following this, the outputs generated were utilized in snippy-core to create a full alignment file. This file was then further refined using snippy-clean_full_aln to obtain a clean full alignment output. Subsequently, this output underwent processing through Gubbins 3.0.0, with the parameter –bootstrap 100, to identify recombination events [60]. To isolate conserved sites from the filtered polymorphic site alignment, we employed the snp-sites tool from Snippy.

### Phylogeny and molecular dating analysis

Construction of a maximum likelihood phylogenetic tree was achieved by leveraging the capabilities of RAxML 8.2.12, invoking the parameters -N autoFC -f a -B 0.03 k -m GTRCAT -x 4743 p 4743 [61]. For temporal analysis, the resultant tree was analysed using the BactDating R package, running for a million iterations [62]. Five *A. baumannii* IC2 strains were used as outgroups for the phylogeny (SAMN02603140, SAMN10716696, SAMEA104305264, SAMN02581277 and SAMN13066390).

Due to variations in sequencing methods and resulting assembly quality, we performed two separate phylogenetic reconstructions. The first included 52 isolates whose assemblies contained fewer than 100 contigs of ≥500 bp, a threshold chosen to ensure high-quality, less fragmented genomes suitable for accurate phylogenetic inference. The second reconstruction included all 60 isolates described in this study, regardless of contig number. To minimize potential biases introduced by lower-quality data, only the first phylogenetic reconstruction was retained.

## Results and discussion

We identified a total of 110 ST158^Pas^ isolates in the literature [12,18, 22,32], from co-authors’ strain collections and/or from the PubMLST (https://pubmlst.org/organisms/acinetobacter-baumannii) and GenBank records (Supplementary Material 1, Table S2). For isolates with a known year of isolation, the earliest was from 2007, and the most recent was from 2022. A large number of the isolates (*n*=100) were collected in or linked to the Middle East (Kuwait, Iraq, Egypt, Saudi Arabia, Lebanon, Jordan and Turkey) or nearby countries (Tunisia, Afghanistan and Pakistan). Ten isolates came from Peru (*n*=4, 2012–2016), China (*n*=2, date of isolation not reported), Belgium (*n*=1, 2014), Russia (*n*=1, 2015), Ireland (*n*=1, 2016) and Indonesia (*n*=1, 2022) without a reported history of international travel or recounted linkage to the Middle East. A connection to a military healthcare base was identified for six isolates. In addition, the four Peruvian and three Jordanian isolates were collected through Naval Medical Research Unit (NAMRU) laboratories, which operate in civilian hospital settings (Table S3). The distribution between countries was skewed by the occurrence of at least two nosocomial outbreaks, accounting for 30 isolates from Kuwait [63] and 7 from Tunisia [24], or by repeated isolation from the same patient, as for the 3 Jordanian isolates (this study).

Of the 110 ST158^Pas^ isolates reported in the literature, we included 60 in our analysis based on the availability of whole-genome sequencing data. We renamed these isolates with the prefix Ab-IC12, followed by the country of isolation and numbered from oldest to newest, with the country of import listed if relevant (Table S3). Of these, 17 isolates had combined short- and long-read sequencing data (Table S4), which allowed 12 isolates to have complete assemblies (fully circularized). Using the Oxford MLST system, 57 isolates belonged to ST499^Oxf^ and 3 to ST1717^Oxf^. The latter were all identified in Egypt. ST499^Oxf^ and ST1717^Oxf^ are single locus variants of each other, the difference being only one nucleotide change in their *rpoD* locus (Supplementary Material 2, Fig. S1).

**Fig. 1.**
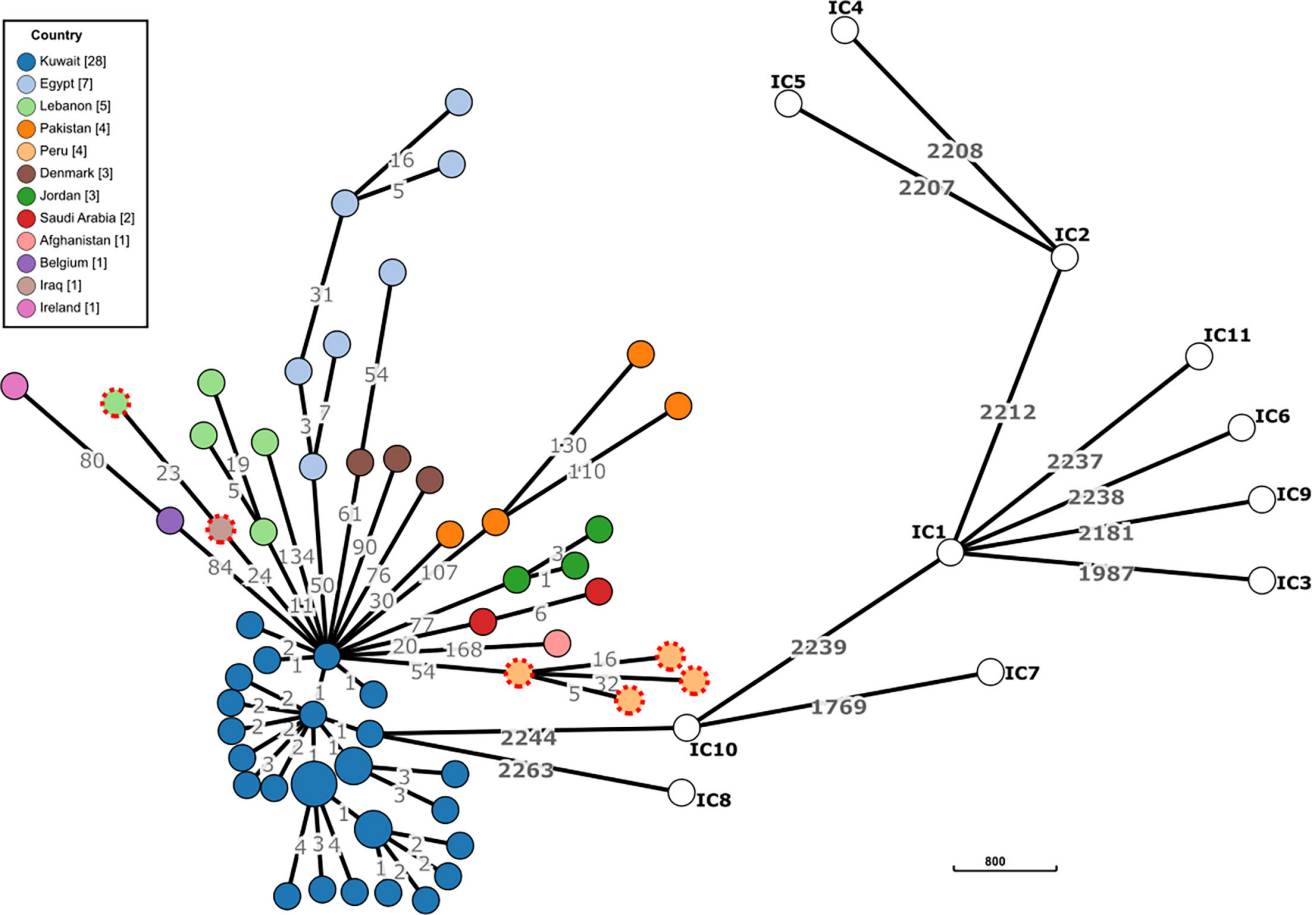
Minimum spanning tree of 60 *A*. *baumannii* IC12 isolates and 11 International Clones (IC1-IC11) based on the cgMLST scheme of Higgins *et al*. [16]. Isolates are colour-coded according to the country of isolation. The length of the branches between isolates is proportional to the log-transformed number of allelic differences (indicated on the branches) based on the cgMLST scheme. All 12 ICs had at least 2,200 allelic differences with ST158^Pas^/IC12. Isolates (*n*=6) with red dotted contours lacked pIC12-2.

Based on cgMLST comparisons, our ST158^Pas^ isolates did not cluster with any previously recognized ICs (Fig. 1). Notably, ST158^Pas^ is very well-differentiated from the known ICs, with over 2,200 allelic differences separating it from IC1-IC11. The Kuwaiti cluster exhibited minimal allelic variation, likely reflecting the nosocomial outbreaks, and may represent a distinct subclone. Given its wide geographical spread throughout North Africa, Asia, Europe and South America, ST158^Pas^ was considered the first ST of a newly designated international clone, initially termed IC10. It is important to note that our reporting of IC10 (ST158^Pas^) was first disseminated as an oral presentation at the April 2023 ECCMID and reported online as a pre-print in May 2023 [23]. However, in August 2023, Shelenkov *et al*. reported that isolates from CC33^Pas^, creating distant and well-formed clusters on the eBURST tree structure of STs of all the *A. baumannii* genomes available in GenBank, would represent a candidate for a new IC, for which the designation IC10 was proposed [18]. To avoid further confusion, we have renamed our previously designated IC10 (ST158^Pas^) as IC12. We aim to clarify this distinction in public databases [25] and future publications. This situation, with two groups nearly simultaneously naming two distinct ICs with the same number, highlights the pressing need for a clear definition of the term ‘IC’ and for a straightforward process of assigning numbers to new ICs. Following such a process will help reduce inconsistencies in future studies [18,23, 25].

All 60 analysed IC12 isolates carried the *ampC-43* allele (100% nucleotide identity), which encoded the *Acinetobacter*-derived cephalosporinase (ADC)-117 variant [26]. In all but one of the 60 isolates, the intrinsic *bla*_OXA-51-like_ gene corresponded to *bla*_OXA-65_. However, as reported by previous studies, the nucleotide sequence of *bla*_OXA-65_ in our isolates showed three silent nucleotide mutations (T90→C, C636→T and A663→G) maintaining 100% amino acid identity compared to the first GenBank-deposited allele of OXA-65 (GenBank: AY750908) [27,28], which was later linked to IC5, corresponding to ST79^Pas^. IC5, which has mostly been found in Latin America [29,31], and our proposed IC12 differed substantially by cgMLST (over 2,000 allele differences), as shown in Fig. 1. This deviation reveals that the current numbering system of the OXAs is probably well-suited for functional comparisons, while it could sometimes be misleading in molecular epidemiological studies [64,65]. In the remaining isolate, Ab-IC12-Peru-2, the encoded OXA-51-like oxacillinase differed by a single amino acid (T74N) compared to the OXA-65 of the other isolates (Supplementary Material 2, Fig. S2). Of note, the *bla*_OXA-65_ gene in one of the previously reported ST158^Pas^ (IC12) isolates from Egypt was disrupted by IS*Aba125* (GenBank: JACSTW000000000.1) [34]. Otherwise, no IS element was found in the vicinity of *ampC-43* or *bla*_OXA-65_ among the 60 isolates analysed in our study.

### Phylogeny and molecular dating analysis

**Fig. 2.**
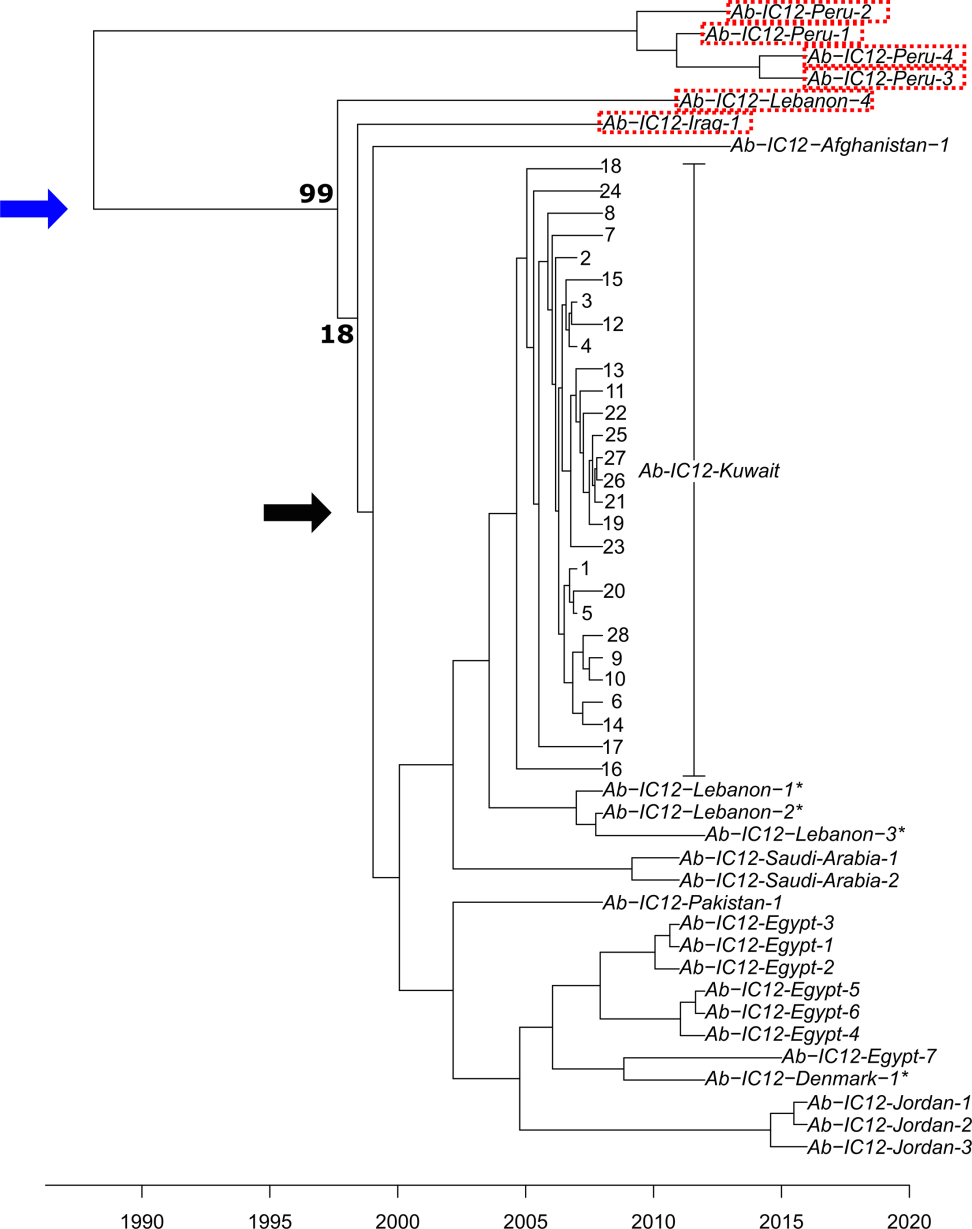
Molecular dating analysis of 52 IC12 isolates. The *x*-axis indicates the estimated timeline of divergence. The blue arrow indicates the proposed acquisition of pIC12-1, present in all isolates except the four from Peru. The black arrow points to the proposed acquisition of pIC12-2. The names of the six isolates lacking pIC12-2 are surrounded by red dots. Asterisks (*) indicate samples from patients with a travel history in another country. Bold numbers indicate bootstrap values.

Based on the phylogenetic tree of 52 IC12 isolates with high-quality sequences, the isolates demonstrated clear grouping by country (Fig. 2). Notably, Ab-IC12-Denmark-1 from a patient with a prior travel history to Egypt clustered with 1 isolate from Egypt, Ab-IC12-Egypt-7 (47 allele differences). One striking finding was the close-to-the-root separation of the four isolates from Peru from all the others. Accordingly, the dissemination from/to Peru most likely took place many years before our earliest detection of IC12, after which the Peruvian isolates followed a different evolutionary path, as denoted by the length of their branch and by their markedly different plasmid content. In addition to containing different combinations of five plasmids not found among any other isolates in this collection (pIC12-6 to pIC12-10), the Peruvian isolates were the only ones lacking pIC12-1.

The phylogenetic tree allowed us to locate a possible single acquisition event of pIC12-1, taking place around 10 years after the split from the Peruvian isolates (Fig. 2). The four Peruvian isolates also lacked pIC12-2, which was also the case for Ab-IC12-Iraq-1 and Ab-IC12-Lebanon-4. The six isolates lacking pIC12-2 appear on branches found on the periphery of the phylogeny. Indeed, the 46 other isolates containing pIC12-2 arose from 1 branch where a single acquisition event of this plasmid can also be proposed (Fig. 2). Following these two single acquisition events, the 46 isolates carrying pIC12-1 and pIC12-2 were split between a core group of 45 isolates clustered by country and the lone isolate from Afghanistan (Ab-IC12-Afghanistan-1).

BactDating [62] allowed us to estimate the time to the most recent common ancestor (MRCA) of IC12, which was 1987 (with a 95% credible interval 1977–1998) with a substitution rate of 1.7 (0.90–2.47) substitutions per genome per year. A possible evolutionary trajectory would be that of an environmental dweller with two notable shifts: (1) transitioning as a human pathogen and (2) acquiring plasmids harbouring antibiotic resistance genes, facilitating their spread in nosocomial settings and their ability to lead to hospital outbreaks as observed in Kuwait in 2007–2008 and in Tunisia in 2008–2009.

All the isolates contained at least one plasmid. However, given that no single plasmid was shared between the Peruvian isolates and the other Middle Eastern/European isolates in our collection, we present the most likely hypothesis that a strain devoid of any plasmid was somehow transferred from the Middle Eastern to Peru (scenario 1) or the other way around (scenario 2). The Middle Eastern and European descendants acquired sequentially pIC12-1 followed by pIC12-2, while the Peruvian lineage’s descendants went on to acquire different plasmids. Another hypothesis, the separate emergence of two IC12-like clones in the Middle East and Peru with no link between them, was considered and rejected based on the results of the cgMLST, the phylogeny and the genomic data.

Arguments in favour of scenario 1 are IC12’s Middle East focus, more specifically its early geographic concentration in several neighbouring countries, the timeline outlined above and the historical link between *A. baumannii* and armed conflict-associated infections in the Middle East [66]. It seems possible that IC12 could have emerged as a successful human pathogen in the context of either of the Iraq wars (the 1991 Gulf War and Operation Iraqi Freedom starting in 2003) or the inter-war period. The latter was marked by a rapidly crumbling healthcare infrastructure in Iraq in the wake of large-scale international sanctions [67,69] affecting all matters of infectious disease practice: access to antibiotics and vaccines, infection control, and wound and surgical care. Its spread in the Middle East and beyond can also be linked in certain cases to military operations or bases, such as in Afghanistan. However, it is important to note that isolates from Jordan and Peru were obtained via the NAMRU, which function as research laboratories collecting samples from civilian hospitals in surrounding areas, not military hospitals or personnel. The molecular dating analysis appears to further support scenario 1, given that the MRCA of the Peruvian isolates appears later than the MRCA of the 48 other isolates. Nevertheless, such an assessment is biassed by the limited number of isolates from Peru, so scenario 2 cannot be excluded. Access to more IC12 isolates, including some predating the earliest ones from 2007, would further help in establishing which of these two scenarios is correct.

### Antimicrobial resistance genes and genetic elements

A total of 26 acquired antimicrobial resistance genes were detected (Table S4): *bla*_OXA-23_, *bla*_GES-11, -12, -22_ and _-35_, *bla*_CARB-16_ and _-49_, *aphA6a*, *aphA1*, *aphA2*, *aacA4*, *aadB* (two different alleles), *strA*, *strB* (two different genes), *cmlA1*, *aadA2b*, *sul1*, *drfA7*, *dfrA1*, *qacE*, *tet*(B), *tet*(X3), *msr*(E), *mph*(E), *bla*_TEM-1B_ and *aacC3*. The Ambler class D ß-lactamase gene *bla*_OXA-23_ was identified in 52/60 isolates. It was located either within Tn*2008* (48 isolates) [70] or Tn*2006* (4 isolates) [71]. In the latter four isolates, Tn*2006* was located in the *A. baumannii* resistance island AbaR4 that was inserted in the chromosomal *comM* gene, as described in isolates from other ICs [72].

A truncated class 1 integron carrying five resistance genes (*aacA4*, *drfA7*, *bla*_GES_, *sul1* and *qacEΔ1*) was surrounded by two miniature inverted-repeat transposable elements (MITEs), forming a 7,486 bp mobile element named MITE*Ab-IC12* (Fig. S3A) [64]. Its insertion created a characteristic target site duplication of 5 bp [73] and was found in 51 isolates. An extended 13,506 bp version, containing five additional resistance genes (*aadB*, *cmlA1*, *aadA2b*, *strA* and *strB*), previously described by Mabrouk *et al*. and Valcek *et al*. [24,36] (GenBank: KY022424.1 and CP102817.1), was detected in three isolates from Lebanon, Ireland and Belgium (Fig. S3B) [24,36]. Four different *bla*_GES_ alleles were detected, which were *bla*_GES-11_ (49 isolates), *bla*_GES-12_ (2 isolates, 1 from Ireland and 1 from Belgium), *bla*_GES-22_ (1 isolate from Denmark with a travel history to Egypt) and _GES-35_ (2 isolates, 1 from Egypt and 1 from Denmark with a travel history to Egypt). A previous study reported the occurrence of another allele, *bla*_GES-14_, in seven IC12 (ST499^Oxf^) isolates from Tunisia [24]. These *bla*_GES_ alleles shared a nucleotide identity of more than 99.7% (862/864) (Fig. S4).

**Fig. 3.**
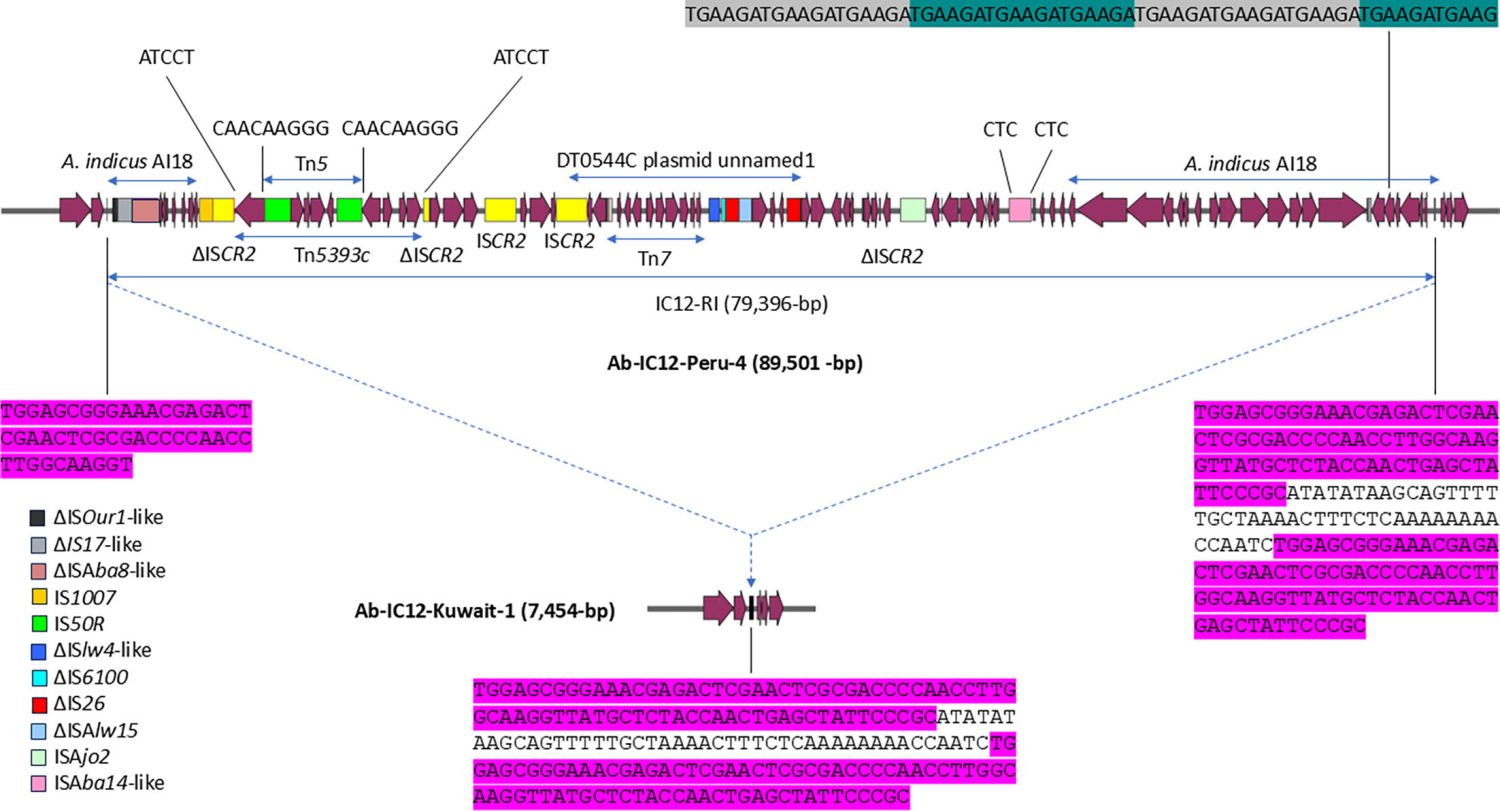
Graphic representation of IC12-RI as present in Ab-IC12-Peru-4’s chromosome. Ab-IC12-Kuwait-1, an IC12-RI-negative isolate, was used to analyse the insertion site. IC12-RI is inserted in a region of two repeated sequences, highlighted in violet. The insertion is associated with a 46 bp duplication. Tn*5*, Tn*5393c*, Tn*7* and regions of high similarity to *A. indicus* AI18 or DT0544C plasmid unnamed1 that are indicated by blue horizontal arrows. Genes and ORFs are shown as plum arrow shapes, with the arrowhead indicating the direction of transcription. Copies of the IS element IS*CR2* are shown as labelled yellow rectangles. Other genes of interest are coloured following the legend on the left. Nucleotide sequences of the target site duplications of Tn*5*, Tn*5393c* and IS*Aba14-like* are indicated in black (not highlighted in any colour), while the nucleotide sequence of a region of three complete and one partial iterons is indicated in black and highlighted in grey and cyan.

The *aphA6a* gene was detected in 49 isolates, exclusively on transposon Tn*aphA6* [17]. As reported by other studies [24,27, 36, 37], Tn*2008*, MITE*Ab-IC12* and Tn*aphA6* coexisted on the same plasmid. The same *aadB* variant (100% nucleotide identity) that was detected in MITE*Ab-IC12* was present in a different genetic context in the chromosome of two isolates from Peru. This chromosomal resistance island, around 79,396 bp long and designated IC12-RI, also carried Tn*5* including *aphA2*, *orf* for bleomycin resistance, and *strB* (GenBank: U00004.1); Tn*5393c* with *strA* and *strB* (GenBank: AF262622.1); IS*CR-2*-related *tet*(X3); IS*CR-2*-related *floR*; IS*CR-2*-related *sul2*; part of Tn*7* with *dfrA1*, *sat2* and *aadA1* (GenBank: KX159450.1 and MN628641.1); *bla*_CARB-16_; and *aadB* with *aphA1* (Fig. 3). Some of the resistance genes within IC12-RI were detected on *A. baumannii* strain DT0544C plasmid unnamed1 (GenBank: CP053216.1) while a large part of the background was similar to the genomic records of *A. indicus* isolates, such as strain AI18 (GenBank: JAAZRX000000000.1) [74]. IC12-RI’s insertion region was characterized by the occurrence of two conserved 76 bp direct repeats that probably played a role in the acquisition of this chromosomal resistance island (Fig. 3).

In our study, the *tet*(X3) gene was present in three isolates from Peru. In two of these isolates, *tet*(X3) resided within the chromosomal IC12-R1 resistance island, unlike its initial identification on a plasmid from *A. junii* Ajun-H1-2 from Israel in 2004 – the earliest known clinical detection [75] – and in *A. baumannii* 34AB, a pig-derived isolate from China in 2017, where three copies of the *tet*(X3) gene were carried on a large plasmid (p34AB, ~300,000 bp) [76]. A related genetic structure to the one found in p34AB was later reported in *A. baumannii* PW12, an environmental isolate from a Ghanaian Tertiary Hospital [77]. The core structure comprising IS*CR2*, Δ*intI* and *tet*(X3) was conserved across p34AB, PW12 and the IC12-RI island in our Peruvian isolates (Ab-IC12-Peru-3 and -4), but the downstream region (~1,100 to 1,200 bp) differed in our isolates, containing *estT*, a serine-dependent macrolide esterase gene [78] also observed downstream of other *tetX* variants, such as *tet*(X5) and *tet*(X6) [79]. Three single-nucleotide differences were noted between *tet*(X3) in p34AB/PW12 and IC12-RI (Fig. S5). For Ab-IC12-Peru-2, the absence of long-read sequencing prevents the determination of the full *tet*(X3)-flanking context.

Another variant of the *aadB* gene was detected on plasmid pRAY* [80] in six isolates from Egypt (*n*=3), Saudi Arabia (*n*=2) and Denmark with a travel history from Egypt (*n*=1). The two *aadB* variants shared a nucleotide identity of 98.9% (Fig. S6). The macrolide resistance *msr*(E)-*mph*(E) operon was detected in 16 isolates. In 15 of these isolates, the operon was surrounded by 2 p*dif* sites, creating a genetic element, called a module, movable by the XerC–XerD system [81]. The *msr*(E)-*mph*(E) module was carried on a repABSDF_p20001-positive plasmid (six isolates from Egypt and two isolates from Denmark, one with a travel history to Egypt and one to Turkey), a *repAci25*-positive plasmid of 13,351 bp (three isolates from Pakistan), a plasmid of 14,584 bp (three isolates from Jordan) or a plasmid of 8,909 bp (one isolate from Peru). Each of these four plasmids had its own replicase gene. In contrast, the *msr*(E)-*mph*(E) operon was most likely located on the chromosome, close to an IS*Ec29* element, in one isolate from Denmark with a travel history to Egypt.

The *bla*_CARB-49_ gene, detected in only two isolates (Ab-IC12-Belgium-1 and Ab-IC12-Ireland-1), encoded an enzyme differing by one amino acid compared to CARB-16. It was surrounded by two copies of IS*Aba1*, forming a 3,499 bp composite transposon. We found two copies of this transposon, provisionally called Tn*CARB-49*, in the genomic records of Ab-IC12-Belgium-1 (AB32-VUB): one located on its chromosome (GenBank: CP091372.1), while the other was plasmid-mediated (pIC12-1e; GenBank: CP102817.1). We were not able to determine the location of Tn*CARB-49* in the other isolate (Ab-IC12-Ireland-1) since we lacked complete genomic data for this strain.

### Plasmids and other mobile genetic elements

Overall, the isolates carried between one and five plasmids, with a total of ten distinct types, named pIC12-1 to pIC12-10, with variants labelled ‘a’ onwards (Table S4) in addition to pRAY* [80]. The most common replicase gene, *rep*_ABSDF_p20001_ (99.66% nucleotide identity to the reference gene ABSDF_p20001, GenBank: CU468232.1), was found in all but four Peruvian isolates. This gene corresponds to group 12 (GR12) in the original *A. baumannii* plasmid typing scheme [56] and to type R3-T10 in the most recent classification [58,82]. Seven variants (pIC12-1a to -1 g, Fig. S7) ranged from 8,251 bp (pIC12-1f) to 22,905 bp (pIC12-1e), sharing a 3,435 bp backbone with five iterons (four complete and one partial, 95 bp).

*repAci6*, the second most common replicase gene (100% nucleotide identity to the *repAci6* gene of pACICU2, GenBank: CP031382.1), was found in most isolates, designated pIC12-2, and linked to group GR6 [56], now type RP-T1 [58]. Its associated plasmid, pIC12-2 (variants pIC12-2a to -2 g), averaged ~80,000 bp and carried a type IV conjugative transfer system (Fig. S8). pIC12-2 was absent in one isolate from Iraq, one from Lebanon and all four from Peru. Partial plasmid sequences excluding the *repAci6* gene were found in one isolate from Lebanon with a history of import from Syria and one isolate from Ireland (Table S4). Resistance elements on pIC12-2 included MITE*Ab-IC12* (or MITE*Ab-IC12-extended*), Tn*2008* and/or Tn*aphA6*, representing the whole array of resistance genes in most of the isolates.

The *repAci25* gene on pIC12-3 was detected in only three Pakistani isolates (Fig. S9), showing 91% nucleotide identity to *repAci4* (GenBank: GU978998.1) as previously reported [25]. *repAci25* corresponds to the R3-T14 [83] and carries the *msr*(E)-*mph*(E) resistance module, as described above. Novel replicase genes were identified, including RepM (WP_019767308.1) on a 5,501 bp plasmid (called pIC12-4) in eight isolates from Egypt or Denmark (imported from Egypt), which lacked antibiotic resistance genes (Fig. S10). Another *rep* gene was found on a 14,584 bp plasmid (pIC12-5) in the three isolates from Jordan (Fig. S11) and one from Saudi Arabia (pMAB25-3; GenBank: CP121593.1), the latter belonging to IC12, was not included in our genomic study.

The remaining five plasmids were uniquely found in Peruvian isolates (Table S4). pIC12-6 (105,850–113,249 bp), which harbours an R3-T3 replicase gene [82,83], was present in all four isolates (Fig. S12), whereas pIC12-7(10,255–16,605 bp) was detected in only two (Fig. S13). A *repAci9-like* gene was found on pIC12-8 in Ab-IC12-Peru-2 isolate (Fig. S14), showing only 85.5% nucleotide identity to the canonical *repAci9* gene, corresponding to GR8 (GenBank: AY541809.1). The original *repAci9* and our *repAci9-*like variant identified here were subsequently assigned to R3-T4 and R3-T13 type, respectively [82]. The backbone of pIC12-8 (8,909 bp) was identical to a plasmid from the *Acinetobacter. lwoffii* strain FDAARGOS (GenBank: CP077374.1). Ab-IC12-Peru-2 also carried a cryptic plasmid, pIC12-9 (2,585 bp) (Fig. S15) and pIC12-10 (~190,000 bp), which resembled pA297-3 (GenBank: KU744946.1). However, the full structure or pIC12-10 remains unresolved.

pIC12-1, pIC12-3, pIC12-5, pIC12-7 and pIC12-8 contained multiple p*dif* sites (Figs S7, S9, S11, S13 and S14), with some p*dif*-flanked modules shuffled across plasmids. For instance, identical recombinase modules were shared by pIC12-1c, pIC12-3 and pIC12-5. However, other modules seemed to have different origins, although they had a similar structure, as noted for the *orf* blue-light sensing using flavin module in pIC12-1e and pIC12-3, sharing <80% nucleotide identity. Evidence of plasmid fusion via two p*dif* sites was suggested by the architecture of pIC12-1d and pIC12-1e, although not experimentally confirmed.

## Conclusions

Fifty-four of the 60 IC12 strains we describe were isolated in or related to the Middle East and neighbouring countries. At least six were linked to military personnel or military healthcare bases. All IC12 isolates carried specific genetic signatures with regard to their OXA-51-like and ADC enzymes (OXA-65 and ADC-117, respectively), and most carried the *bla*_OXA-23_ carbapenemase gene. Based on their plasmid content, the evolutionary pathway of the 4 strains isolated in Peru was strikingly different from that of the 56 other strains we analysed. As prevention and treatment of infections with *A. baumannii* are increasingly challenging and eradication of hospital-adapted strains is commonly difficult, primarily in low- and middle-income countries, there is a pressing need to expand surveillance to facilitate the detection of novel clones, resistance genes and mobile genetic elements in human and non-human associated settings [84]. We hope this study and other recent multinational collaborative investigations [23,25] represent the beginning of much-needed efforts to expand our understanding of the growing diversity observed among *A. baumannii* isolates.

## Supplementary material

10.1099/mgen.0.001572 Supplementary Material 1.

10.1099/mgen.0.001572 Supplementary Material 2.

## Abbreviations

ADC: *Acinetobacter*-derived cephalosporinase
CC: clonal complex
cgMLST: core genome multilocus sequence typing
CRAB: carbapenem-resistant *Acinetobacter baumannii*
IC: International Clone
IS: insertion sequence
MITEs: miniature inverted-repeat transposable elements
MLST: multilocus sequence typing
MRCA: most recent common ancestor
NAMRU: Naval Medical Research Unit
NEB: New England Biolabs
ONT: Oxford Nanopore Technologies
Oxf: Oxford
Pas: Pasteur
ST: sequence type
UdS: Université de Sherbrooke
