# Supplementary material for: Global emergence of Acinetobacter baumannii International Clone 12 predominantly found in the Middle East
Source: Microb Genom. 2025 Nov 28;11(11):001572. doi: 10.1099/mgen.0.001572 (PMC12662599; doi:10.1099/mgen.0.001572)
Supplement: Supplementary Material 2. [file mgen-11-01572-s002.pdf]

Supplementary Figure S1.

>*rpoD*-26 (ST499^{Oxf})

```
TGGCCAAATGCTGTTGAAGTTGTATTAAAAGAATATAATGATTTTTTAACTGGCGAACGCCGTCTTGCCGATATCTTATCTGGTTATTTAGACC
CTGAAACTGATGAAGATATTCCTGAAGTTTTAGAAGACGTTGAAGAACTTGAGGAAGAAGACGAATCATCTACAAAGTCAACGAAAGAAGTTAA
GCTTGATGATGACGATGAAGAAGAAGAGTCTGAAGGTGATGATGATTCTGAAGGTGATTCAGGTCCAGACCCTGAAGTTGCAAAAGTTCGTTTT
GCTGAATTAGAAGCTGCATGGGCTCAAACTAAAGCTGTCATCGAAAAACATGGCCGTAATAGCCCTGAAGCAAATGAAGCTCTTGAGTCTTTAG
CAACTGTATTTATGATGTTTAAATTTACTCCACGTTTATTTGACATCATTTCAGAAATGATTCGCGGAACTCATGAACAAATTCGTGCCAATGA
ACGTGAAATTATGCGTTATGCAGTACGTCGTGGCCGTATGGAC
```

>*rpoD*-141 (ST1717^{Oxf})

```
TGGCCAAATGCTGTTGAAGTTGTATTAAAAGAATATAATGATTTTTTAACTGGCGAACGCCGTCTTGCCGATATCTTATCTGGTTATTTAGACC
CTGAAACTGATGAAGATATTCCTGAAGTTTTAGAAGACGTTGAAGAACTTGAGGAAGAAGACGAATCATCTACAAAGTCAACGAAAGAAGTTAA
GCTTGATGATGACGATGAAGAAGAAGAGTCTGAAGGTGATGATGATTCTGAAGGTGATTCAGGTCCAGACCCTGAAGTTGCAAAAGTTCGTTTT
GCTGAATTAGAAGCTGCATGGGCTCAAACTAAAGCTGTCATCGAAAAACATGGCCGTAATAGCCCTGAAGCAAATGAAGCTTTTGAGTCTTTAG
CAACTGTATTTATGATGTTTAAATTTACTCCACGTTTATTTGACATCATTTCAGAAATGATTCGCGGAACTCATGAACAAATTCGTGCCAATGA
ACGTGAAATTATGCGTTATGCAGTACGTCGTGGCCGTATGGAC
```

```
rpoD-26       TGGCCAAATGCTGTTGAAGTTGTATTAAAAGAATATAATGATTTTTTAACTGGCGAACGC 60
rpoD-141      TGGCCAAATGCTGTTGAAGTTGTATTAAAAGAATATAATGATTTTTTAACTGGCGAACGC 60
              ************************************************************

rpoD-26       CGTCTTGCCGATATCTTATCTGGTTATTTAGACCCTGAAACTGATGAAGATATTCCTGAA 120
rpoD-141      CGTCTTGCCGATATCTTATCTGGTTATTTAGACCCTGAAACTGATGAAGATATTCCTGAA 120
              ************************************************************

rpoD-26       GTTTTAGAAGACGTTGAAGAACTTGAGGAAGAAGACGAATCATCTACAAAGTCAACGAAA 180
rpoD-141      GTTTTAGAAGACGTTGAAGAACTTGAGGAAGAAGACGAATCATCTACAAAGTCAACGAAA 180
              ************************************************************

rpoD-26       GAAGTTAAGCTTGATGATGACGATGAAGAAGAAGAGTCTGAAGGTGATGATGATTCTGAA 240
rpoD-141      GAAGTTAAGCTTGATGATGACGATGAAGAAGAAGAGTCTGAAGGTGATGATGATTCTGAA 240
              ************************************************************

rpoD-26       GGTGATTCAGGTCCAGACCCTGAAGTTGCAAAAGTTCGTTTTGCTGAATTAGAAGCTGCA 300
rpoD-141      GGTGATTCAGGTCCAGACCCTGAAGTTGCAAAAGTTCGTTTTGCTGAATTAGAAGCTGCA 300
              ************************************************************

rpoD-26       TGGGCTCAAACTAAAGCTGTCATCGAAAAACATGGCCGTAATAGCCCTGAAGCAAATGAA 360
rpoD-141      TGGGCTCAAACTAAAGCTGTCATCGAAAAACATGGCCGTAATAGCCCTGAAGCAAATGAA 360
              ************************************************************

rpoD-26       GCTCTTGAGTCTTTAGCAACTGTATTTATGATGTTTAAATTTACTCCACGTTTATTTGAC 420
rpoD-141      GCTTTTGAGTCTTTAGCAACTGTATTTATGATGTTTAAATTTACTCCACGTTTATTTGAC 420
              *** ********************************************************

rpoD-26       ATCATTTCAGAAATGATTCGCGGAACTCATGAACAAATTCGTGCCAATGAACGTGAAATT 480
rpoD-141      ATCATTTCAGAAATGATTCGCGGAACTCATGAACAAATTCGTGCCAATGAACGTGAAATT 480
              ************************************************************

rpoD-26       ATGCGTTATGCAGTACGTCGTGGCCGTATGGAC      513
rpoD-141      ATGCGTTATGCAGTACGTCGTGGCCGTATGGAC      513
              *********************************
```

Nucleotide sequence alignment of the *rpoD* alleles 26 (sequence type 499) and 141 (sequence type 1717) according to the Oxford scheme for multilocus sequence typing of *Acinetobacter baumannii*. One single nucleotide change was detected (highlighted in yellow). The alignment was done using Clustal Omega (https://www.ebi.ac.uk/Tools/msa/clustalo/).

Supplementary Figure S2.

```
>OXA-65 (Ab-IC12-Kuwait-1)
MNIKALLLITSAIFISACSPYIVTANPNHSASKSDEKAEKIKNLFNEAHTTGVLVIQQGQTQQSYGNDLARASTEYVPASTFKMLNALIGLEHH
KATTTEVFKWDGKKRLFPEWEKDMTLGDAMKASAIPVYQDLARRIGLELMSKEVKRVGYGNADIGTQVDNFWLVGPLKITPQQEAQFAYKLANK
TLPFSQKVQDEVQSMLFIEEKNGNKIYAKSGWGWDVNPQVGWLTGWVVQPQGNIVAFSLNLEMKKGIPSSVRKEITYKSLEQLGIL

>OXA-65-like (Ab-IC12-Peru-2)
MNIKALLLITSAIFISACSPYIVTANPNHSASKSDEKAEKIKNLFNEAHTTGVLVIQQGQTQQSYGNDLARASNEYVPASTFKMLNALIGLEHH
KATTTEVFKWDGKKRLFPEWEKDMTLGDAMKASAIPVYQDLARRIGLELMSKEVKRVGYGNADIGTQVDNFWLVGPLKITPQQEAQFAYKLANK
TLPFSQKVQDEVQSMLFIEEKNGNKIYAKSGWGWDVNPQVGWLTGWVVQPQGNIVAFSLNLEMKKGIPSSVRKEITYKSLEQLGIL

OXA-65          MNIKALLLITSAIFISACSPYIVTANPNHSASKSDEKAEKIKNLFNEAHTTGVLVIQQGQ         60
OXA-65-like     MNIKALLLITSAIFISACSPYIVTANPNHSASKSDEKAEKIKNLFNEAHTTGVLVIQQGQ         60
                ************************************************************

OXA-65          TQQSYGNDLARASTEYVPASTFKMLNALIGLEHHKATTTEVFKWDGKKRLFPEWEKDMTL         120
OXA-65-like     TQQSYGNDLARASNEYVPASTFKMLNALIGLEHHKATTTEVFKWDGKKRLFPEWEKDMTL         120
                *************.**********************************************

OXA-65          GDAMKASAIPVYQDLARRIGLELMSKEVKRVGYGNADIGTQVDNFWLVGPLKITPQQEAQ         180
OXA-65-like     GDAMKASAIPVYQDLARRIGLELMSKEVKRVGYGNADIGTQVDNFWLVGPLKITPQQEAQ         180
                ************************************************************

OXA-65          FAYKLANKTLPFSQKVQDEVQSMLFIEEKNGNKIYAKSGWGWDVNPQVGWLTGWVVQPQG         240
OXA-65-like     FAYKLANKTLPFSQKVQDEVQSMLFIEEKNGNKIYAKSGWGWDVNPQVGWLTGWVVQPQG         240
                ************************************************************

OXA-65          NIVAFSLNLEMKKGIPSSVRKEITYKSLEQLGIL      274
OXA-65-like     NIVAFSLNLEMKKGIPSSVRKEITYKSLEQLGIL      274
                **********************************
```

Amino acid sequence alignment of OXA-65 (encoded in the genomes of all our isolates except for Ab-IC12-Peru-2) and OXA-65-like (encoded in the genome of Ab-IC12-Peru-2). One amino acid change (T74N) was detected (highlighted in yellow). The alignment was done using Clustal Omega (https://www.ebi.ac.uk/Tools/msa/clustalo/).

Supplementary Figure S3.

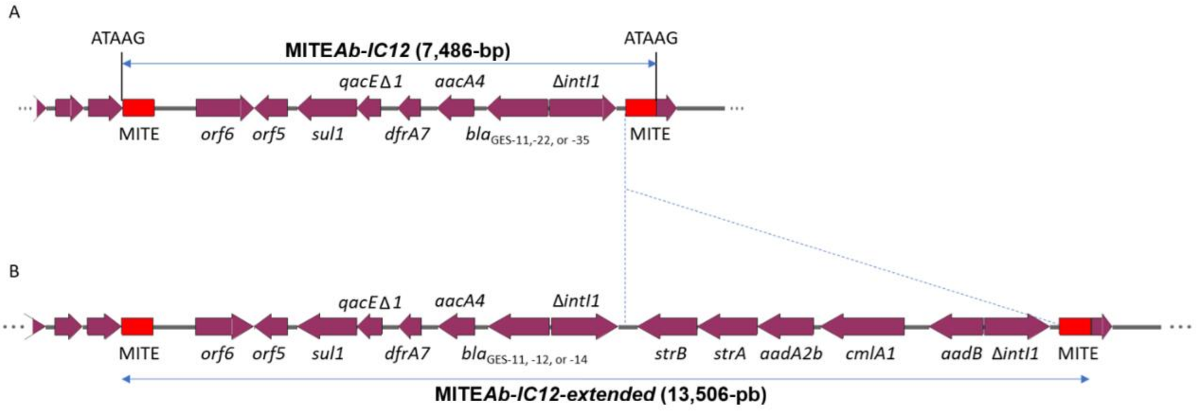

Graphic representation of MITE*Ab-IC12* and MITE*Ab-IC12*-extended as present in Ab-IC12-Kuwait-1 (A) and Ab-IC12-Belgium-1 (B). MITE*Ab-IC12* and MITE*Ab-IC12-extended* comprised several resistance genes surrounded by two Miniature Inverted-repeat Transposable Elements (MITEs), shown as labeled red rectangles. Genes and open reading frames (*orf*) were shown as plum arrow shapes, with the arrowhead indicating the direction of transcription. The insertion of MITE*Ab-IC12* and MITE*Ab-IC12-extended* was associated with a target site duplication of 5-bp (ATAAG).

Supplementary Figure S4.

A

>*bla*$_{\text{GES-11}}$

ATGCGCTTCATTCACGCACTATTACTGGCAGGGATCGCTCACTCTGCATATGCGTCGGAAAAATTAACCTTCAAGACCGATCTTGAGAAGCTAG
AGCGCGAAAAAGCAGCTCAGATCGGTGTTGCGATCGTCGATCCCCAAGGAGAGATCGTCGCGGGCCACCGAATGGCGCAGCGTTTTGCAATGTG
CTCAACGTTCAAGTTTCCGCTAGCCGCGCTGGTCTTTGAAAGAATTGACTCAGGCACCGAGCGGGGGGATCGAAAACTTTCATATGGGCCGGAC
ATGATCGTCGAATGGTCTCCTGCCACGGAGCGGTTTCTAGCATCGGGACACATGACGGTTCTCGAGGCAGCGCAAGCTGCGGTGCAGCTTAGCG
ACAATGGGGCTACTAACCTCTTACTGAGAGAAATTGGCGGACCTGCTGCAATGACGCAGTATTTTCGTAAAATTGGCGACTCTGTGAGTCGGCT
AGACCGGAAAGAGCCGGAGATGGGCGACAACACACCTGGCGACCTCAGAGATACAACTACGCCTATTGCTATGGCACGTACTGTGGCTAAAGTC
CTCTATGGCGGCGCACTGACGTCCACCTCGACCCACACCATTGAGAGGTGGCTGATCGGAAACCAAACGGGAGACGCGACACTACGAGCGGGTT
TTCCTAAAGATTGGGTTGTTGGAGAGAAAACTGGTACCTGCGCCAACGGGGCCCGGAACGACATTGGTTTTTTAAAGCCCAGGAGAGAGATTA
CGCTGTAGCGGTGTATACAACGGCCCCGAAACTATCGGCCGTAGAACGTGACGAATTAGTTGCCTCTGTCGGTCAAGTTATTACACAACTCATC
CTGAGCACGGACAAATAG

>*bla*$_{\text{GES-12}}$

ATGCGCTTCATTCACGCACTATTACTGGCAGGGATCGCTCACTCTGCATATGCGTCGGAAAAATTAACCTTCAAGACCGATCTTGAGAAGCTAG
AGCGCGAAAAAGCAGCTCAGATCGGTGTTGCGATCGTCGATCCCCAAGGAGAGATCGTCGCGGGCCACCGAATGGCGCAGCGTTTTGCAATGTG
CTCAACGTTCAAGTTTCCGCTAGCCGCGCTGGTCTTTGAAAGAATTGACTCAGGCACCGAGCGGGGGGATCGAAAACTTTCATATGGGCCGGAC
ATGATCGTCGAATGGTCTCCTGCCACGGAGCGGTTTCTAGCATCGGGACACATGACGGTTCTCGAGGCAGCGCAAGCTGCGGTGCAGCTTAGCG
ACAATGGGGCTACTAACCTCTTACTGAGAGAAATTGGCGGACCTGCTGCAATGACGCAGTATTTTCGTAAAATTGGCGACTCTGTGAGTCGGCT
AGACCGGAAAGAGCCGGAGATGGGCGACAACACACCTGGCGACCTCAGAGATACAACTACGCCTATTGCTATGGCACGTACTGTGGCTAAAGTC
CTCTATGGCGGCGCACTGACGTCCACCTCGACCCACACCATTGAGAGGTGGCTGATCGGAAACCAAACGGGAGACGCGACACTACGAGCGGGTT
TTCCTAAAGATTGGGTTGTTGGAGAGAAAACTGGTGCCTGCGCCAACGGGGCCCGGAACGACATTGGTTTTTTAAAGCCCAGGAGAGAGATTA
CGCTGTAGCGGTGTATACAACGGCCCCGAAACTATCGGCCGTAGAACGTGACGAATTAGTTGCCTCTGTCGGTCAAGTTATTACACAACTCATC
CTGAGCACGGACAAATAG

>*bla*$_{\text{GES-22}}$

ATGCGCTTCATTCACGCACTATTACTGGCAGGGATCGCTCACTCTGCATATGCGTCGGAAAAATTAACCTTCAAGACCGATCTTGAGAAGCTAG
AGCGCGAAAAAGCAGCTCAGATCGGTGTTGCGATCGTCGATCCCCAAGGAGAGATCGTCGCGGGCCACCGAATGGCGCAGCGTTTTGCAATGTG
CTCAACGTTCAAGTTTCCGCTAGCCGCGCTGGTCTTTGAAAGAATTGACTCAGGCACCGAGCGGGGGGATCGAAAACTTTCATATGGGCCGGAC
ATGATCGTCGAATGGTCTCCTGCCACGGAGCGGTTTCTAGCATCGGGACACATGACGGTTCTCGAGGCAGCGCAAGCTGCGGTGCAGCTTAGCG
ACAATGGGGCTACTAACCTCTTACTGAGAGAAATTGGCGGACCTGCTGCAATGACGCAGTATTTTCGTAAAATTGGCGACTCTGTGAGTCGGCT
AGACCGGAAAGAGCCGGAGTTGGGCGACAACACACCTGGCGACCTCAGAGATACAACTACGCCTATTGCTATGGCACGTACTGTGGCTAAAGTC
CTCTATGGCGGCGCACTGACGTCCACCTCGACCCACACCATTGAGAGGTGGCTGATCGGAAACCAAACGGGAGACGCGACACTACGAGCGGGTT
TTCCTAAAGATTGGGTTGTTGGAGAGAAAACTGGTACCTGCGCCAACGGGGCCCGGAACGACATTGGTTTTTTAAAGCCCAGGAGAGAGATTA
CGCTGTAGCGGTGTATACAACGGCCCCGAAACTATCGGCCGTAGAACGTGACGAATTAGTTGCCTCTGTCGGTCAAGTTATTACACAACTCATC
CTGAGCACGGACAAATAG

>*bla*$_{\text{GES-35}}$

ATGCGCTTCATTCACGCACTATTACTGGCAGGGATCGCTCACTCTGCATATGCGTCGGAAAAATTAACCTTCAAGACCGATCTTGAGAAGCTAG
AGCGCGAAAAAGCAGCTCAGATCGGTGTTGCGATCGTCGATCCCCAAGGAGAGATCGTCGCGGGCCACCGAATGGCGCAGCGTTTTGCAATGTG
CTCAACGTTCAAGTTTCCGCTAGCCGCGCTGGTCTTTGAAAGAATTGACTCAGGCACCGAGCGGGGGGATCGAAAACTTTCATATGGGCCGGAC
ATGATCGTCGAATGGTCTCCTGCCACGGAGCGGTTTCTAGCATCGGGACACATGACGGTTCTCGAGGCAGCGCAAGCTGCGGTGCAGCTTAGCG
ACAATGGGGCTACTAACCTCTTACTGAGAGAAATTGGCGGACCTGCTGCAATGACGCAGTATTTTCGTAAAATTGGCGACTCTGTGAGTCGGCT
AGACCGGAAAGAGCCGGAGTTGGGCGACAACACACCTGGCGACCTCAGAGATACAACTACGCCTATTGCTATGGCACGTACTGTGGCTAAAATC
CTCTATGGCGGCGCACTGACGTCCACCTCGACCCACACCATTGAGAGGTGGCTGATCGGAAACCAAACGGGAGACGCGACACTACGAGCGGGTT
TTCCTAAAGATTGGGTTGTTGGAGAGAAAACTGGTACCTGCGCCAACGGGGCCCGGAACGACATTGGTTTTTTAAAGCCCAGGAGAGAGATTA
CGCTGTAGCGGTGTATACAACGGCCCCGAAACTATCGGCCGTAGAACGTGACGAATTAGTTGCCTCTGTCGGTCAAGTTATTACACAACTCATC
CTGAGCACGGACAAATAG

```
blaGES-35      ATGCGCTTCATTCACGCACTATTACTGGCAGGGATCGCTCACTCTGCATATGCGTCGGAA  60
blaGES-22      ATGCGCTTCATTCACGCACTATTACTGGCAGGGATCGCTCACTCTGCATATGCGTCGGAA  60
blaGES-11      ATGCGCTTCATTCACGCACTATTACTGGCAGGGATCGCTCACTCTGCATATGCGTCGGAA  60
blaGES-12      ATGCGCTTCATTCACGCACTATTACTGGCAGGGATCGCTCACTCTGCATATGCGTCGGAA  60
               ************************************************************

blaGES-35      AAATTAACCTTCAAGACCGATCTTGAGAAGCTAGAGCGCGAAAAAGCAGCTCAGATCGGT  120
blaGES-22      AAATTAACCTTCAAGACCGATCTTGAGAAGCTAGAGCGCGAAAAAGCAGCTCAGATCGGT  120
blaGES-11      AAATTAACCTTCAAGACCGATCTTGAGAAGCTAGAGCGCGAAAAAGCAGCTCAGATCGGT  120
blaGES-12      AAATTAACCTTCAAGACCGATCTTGAGAAGCTAGAGCGCGAAAAAGCAGCTCAGATCGGT  120
               ************************************************************

blaGES-35      GTTGCGATCGTCGATCCCCAAGGAGAGATCGTCGCGGGCCACCGAATGGCGCAGCGTTTT  180
blaGES-22      GTTGCGATCGTCGATCCCCAAGGAGAGATCGTCGCGGGCCACCGAATGGCGCAGCGTTTT  180
blaGES-11      GTTGCGATCGTCGATCCCCAAGGAGAGATCGTCGCGGGCCACCGAATGGCGCAGCGTTTT  180
blaGES-12      GTTGCGATCGTCGATCCCCAAGGAGAGATCGTCGCGGGCCACCGAATGGCGCAGCGTTTT  180
               ************************************************************

blaGES-35      GCAATGTGCTCAACGTTCAAGTTTCCGCTAGCCGCGCTGGTCTTTGAAAGAATTGACTCA  240
blaGES-22      GCAATGTGCTCAACGTTCAAGTTTCCGCTAGCCGCGCTGGTCTTTGAAAGAATTGACTCA  240
blaGES-11      GCAATGTGCTCAACGTTCAAGTTTCCGCTAGCCGCGCTGGTCTTTGAAAGAATTGACTCA  240
blaGES-12      GCAATGTGCTCAACGTTCAAGTTTCCGCTAGCCGCGCTGGTCTTTGAAAGAATTGACTCA  240
               ************************************************************

blaGES-35      GGCACCGAGCGGGGGGATCGAAAACTTTCATATGGGCCGGACATGATCGTCGAATGGTCT  300
blaGES-22      GGCACCGAGCGGGGGGATCGAAAACTTTCATATGGGCCGGACATGATCGTCGAATGGTCT  300
```

```
blaGES-11      GGCACCGAGCGGGGGGATCGAAAACTTTCATATGGGCCGGACATGATCGTCGAATGGTCT  300
blaGES-12      GGCACCGAGCGGGGGGATCGAAAACTTTCATATGGGCCGGACATGATCGTCGAATGGTCT  300
               ************************************************************

blaGES-35      CCTGCCACGGAGCGGTTTCTAGCATCGGGACACATGACGGTTCTCGAGGCAGCGCAAGCT  360
blaGES-22      CCTGCCACGGAGCGGTTTCTAGCATCGGGACACATGACGGTTCTCGAGGCAGCGCAAGCT  360
blaGES-11      CCTGCCACGGAGCGGTTTCTAGCATCGGGACACATGACGGTTCTCGAGGCAGCGCAAGCT  360
blaGES-12      CCTGCCACGGAGCGGTTTCTAGCATCGGGACACATGACGGTTCTCGAGGCAGCGCAAGCT  360
               ************************************************************

blaGES-35      GCGGTGCAGCTTAGCGACAATGGGGCTACTAACCTCTTACTGAGAGAAATTGGCGGACCT  420
blaGES-22      GCGGTGCAGCTTAGCGACAATGGGGCTACTAACCTCTTACTGAGAGAAATTGGCGGACCT  420
blaGES-11      GCGGTGCAGCTTAGCGACAATGGGGCTACTAACCTCTTACTGAGAGAAATTGGCGGACCT  420
blaGES-12      GCGGTGCAGCTTAGCGACAATGGGGCTACTAACCTCTTACTGAGAGAAATTGGCGGACCT  420
               ************************************************************

blaGES-35      GCTGCAATGACGCAGTATTTTCGTAAAATTGGCGACTCTGTGAGTCGGCTAGACCGGAAA  480
blaGES-22      GCTGCAATGACGCAGTATTTTCGTAAAATTGGCGACTCTGTGAGTCGGCTAGACCGGAAA  480
blaGES-11      GCTGCAATGACGCAGTATTTTCGTAAAATTGGCGACTCTGTGAGTCGGCTAGACCGGAAA  480
blaGES-12      GCTGCAATGACGCAGTATTTTCGTAAAATTGGCGACTCTGTGAGTCGGCTAGACCGGAAA  480
               ************************************************************

blaGES-35      GAGCCGGAGTTGGGCGACAACACACCTGGCGACCTCAGAGATACAACTACGCCTATTGCT  540
blaGES-22      GAGCCGGAGTTGGGCGACAACACACCTGGCGACCTCAGAGATACAACTACGCCTATTGCT  540
blaGES-11      GAGCCGGAGATGGGCGACAACACACCTGGCGACCTCAGAGATACAACTACGCCTATTGCT  540
blaGES-12      GAGCCGGAGATGGGCGACAACACACCTGGCGACCTCAGAGATACAACTACGCCTATTGCT  540
               ********* **************************************************

blaGES-35      ATGGCACGTACTGTGGCTAAAATCCTCTATGGCGGCGCACTGACGTCCACCTCGACCCAC  600
blaGES-22      ATGGCACGTACTGTGGCTAAAGTCCTCTATGGCGGCGCACTGACGTCCACCTCGACCCAC  600
blaGES-11      ATGGCACGTACTGTGGCTAAAGTCCTCTATGGCGGCGCACTGACGTCCACCTCGACCCAC  600
blaGES-12      ATGGCACGTACTGTGGCTAAAGTCCTCTATGGCGGCGCACTGACGTCCACCTCGACCCAC  600
               ********************* **************************************

blaGES-35      ACCATTGAGAGGTGGCTGATCGGAAACCAAACGGGAGACGCGACACTACGAGCGGGTTTT  660
blaGES-22      ACCATTGAGAGGTGGCTGATCGGAAACCAAACGGGAGACGCGACACTACGAGCGGGTTTT  660
blaGES-11      ACCATTGAGAGGTGGCTGATCGGAAACCAAACGGGAGACGCGACACTACGAGCGGGTTTT  660
blaGES-12      ACCATTGAGAGGTGGCTGATCGGAAACCAAACGGGAGACGCGACACTACGAGCGGGTTTT  660
               ************************************************************

blaGES-35      CCTAAAGATTGGGTTGTTGGAGAGAAAACTGGTACCTGCGCCAACGGGGCCCGGAACGAC  720
blaGES-22      CCTAAAGATTGGGTTGTTGGAGAGAAAACTGGTACCTGCGCCAACGGGGCCCGGAACGAC  720
blaGES-11      CCTAAAGATTGGGTTGTTGGAGAGAAAACTGGTACCTGCGCCAACGGGGCCCGGAACGAC  720
blaGES-12      CCTAAAGATTGGGTTGTTGGAGAGAAAACTGGTGCCTGCGCCAACGGGGCCCGGAACGAC  720
               ********************************* **************************

blaGES-35      ATTGGTTTTTTTAAAGCCCAGGAGAGAGATTACGCTGTAGCGGTGTATACAACGGCCCCG  780
blaGES-22      ATTGGTTTTTTTAAAGCCCAGGAGAGAGATTACGCTGTAGCGGTGTATACAACGGCCCCG  780
blaGES-11      ATTGGTTTTTTTAAAGCCCAGGAGAGAGATTACGCTGTAGCGGTGTATACAACGGCCCCG  780
blaGES-12      ATTGGTTTTTTTAAAGCCCAGGAGAGAGATTACGCTGTAGCGGTGTATACAACGGCCCCG  780
               ************************************************************

blaGES-35      AAACTATCGGCCGTAGAACGTGACGAATTAGTTGCCTCTGTCGGTCAAGTTATTACACAA  840
blaGES-22      AAACTATCGGCCGTAGAACGTGACGAATTAGTTGCCTCTGTCGGTCAAGTTATTACACAA  840
blaGES-11      AAACTATCGGCCGTAGAACGTGACGAATTAGTTGCCTCTGTCGGTCAAGTTATTACACAA  840
blaGES-12      AAACTATCGGCCGTAGAACGTGACGAATTAGTTGCCTCTGTCGGTCAAGTTATTACACAA  840
               ************************************************************

blaGES-35      CTCATCCTGAGCACGGACAAATAG         864
blaGES-22      CTCATCCTGAGCACGGACAAATAG         864
blaGES-11      CTCATCCTGAGCACGGACAAATAG         864
blaGES-12      CTCATCCTGAGCACGGACAAATAG         864
               ************************
```

B

```
>GES-11
MRFIHALLLAGIAHSAYASEKLTFKTDLEKLEREKAAQIGVAIVDPQGEIVAGHRMAQRFAMCSTFKFPLAALVFERIDSGTERGDRKLSYGPD
MIVEWSPATERFLASGHMTVLEAAQAAVQLSDNGATNLLLREIGGPAAMTQYFRKIGDSVSRLDRKEPEMGDNTPGDLRDTTTPIAMARTVAKV
LYGGALTSTSTHTIERWLIGNQTGDATLRAGFPKDWVVGEKTGTCANGARNDIGFFKAQERDYAVAVYTTAPKLSAVERDELVASVGQVITQLI
LSTDK

>GES-12
MRFIHALLLAGIAHSAYASEKLTFKTDLEKLEREKAAQIGVAIVDPQGEIVAGHRMAQRFAMCSTFKFPLAALVFERIDSGTERGDRKLSYGPD
MIVEWSPATERFLASGHMTVLEAAQAAVQLSDNGATNLLLREIGGPAAMTQYFRKIGDSVSRLDRKEPEMGDNTPGDLRDTTTPIAMARTVAKV
LYGGALTSTSTHTIERWLIGNQTGDATLRAGFPKDWVVGEKTGACANGARNDIGFFKAQERDYAVAVYTTAPKLSAVERDELVASVGQVITQLI
LSTDK

>GES-22
```

```
MRFIHALLLAGIAHSAYASEKLTFKTDLEKLEREKAAQIGVAIVDPQGEIVAGHRMAQRFAMCSTFKFPLAALVFERIDSGTERGDRKLSYGPD
MIVEWSPATERFLASGHMTVLEAAQAAVQLSDNGATNLLLREIGGPAAMTQYFRKIGDSVSRLDRKEPELGDNTPGDLRDTTTPIAMARTVAKV
LYGGALTSTSTHTIERWLIGNQTGDATLRAGFPKDWVVGEKTGTCANGARNDIGFFKAQERDYAVAVYTTAPKLSAVERDELVASVGQVITQLI
LSTDK

>GES-35
MRFIHALLLAGIAHSAYASEKLTFKTDLEKLEREKAAQIGVAIVDPQGEIVAGHRMAQRFAMCSTFKFPLAALVFERIDSGTERGDRKLSYGPD
MIVEWSPATERFLASGHMTVLEAAQAAVQLSDNGATNLLLREIGGPAAMTQYFRKIGDSVSRLDRKEPELGDNTPGDLRDTTTPIAMARTVAKI
LYGGALTSTSTHTIERWLIGNQTGDATLRAGFPKDWVVGEKTGTCANGARNDIGFFKAQERDYAVAVYTTAPKLSAVERDELVASVGQVITQLI
LSTDK
```

File S1 (D). Amino acid changes (highlighted in red) in GES-12, GES-22, and GES-35 in reference to GES-11 as shown by Clustal Omega (https://www.ebi.ac.uk/Tools/msa/clustalo/)

```
GES-35      MRFIHALLLAGIAHSAYASEKLTFKTDLEKLEREKAAQIGVAIVDPQGEIVAGHRMAQRF    60
GES-22      MRFIHALLLAGIAHSAYASEKLTFKTDLEKLEREKAAQIGVAIVDPQGEIVAGHRMAQRF    60
GES-11      MRFIHALLLAGIAHSAYASEKLTFKTDLEKLEREKAAQIGVAIVDPQGEIVAGHRMAQRF    60
GES-12      MRFIHALLLAGIAHSAYASEKLTFKTDLEKLEREKAAQIGVAIVDPQGEIVAGHRMAQRF    60
            ************************************************************

GES-35      AMCSTFKFPLAALVFERIDSGTERGDRKLSYGPDMIVEWSPATERFLASGHMTVLEAAQA    120
GES-22      AMCSTFKFPLAALVFERIDSGTERGDRKLSYGPDMIVEWSPATERFLASGHMTVLEAAQA    120
GES-11      AMCSTFKFPLAALVFERIDSGTERGDRKLSYGPDMIVEWSPATERFLASGHMTVLEAAQA    120
GES-12      AMCSTFKFPLAALVFERIDSGTERGDRKLSYGPDMIVEWSPATERFLASGHMTVLEAAQA    120
            ************************************************************

GES-35      AVQLSDNGATNLLLREIGGPAAMTQYFRKIGDSVSRLDRKEPELGDNTPGDLRDTTTPIA    180
GES-22      AVQLSDNGATNLLLREIGGPAAMTQYFRKIGDSVSRLDRKEPELGDNTPGDLRDTTTPIA    180
GES-11      AVQLSDNGATNLLLREIGGPAAMTQYFRKIGDSVSRLDRKEPEMGDNTPGDLRDTTTPIA    180
GES-12      AVQLSDNGATNLLLREIGGPAAMTQYFRKIGDSVSRLDRKEPEMGDNTPGDLRDTTTPIA    180
            *******************************************:****************

GES-35      MARTVAKILYGGALTSTSTHTIERWLIGNQTGDATLRAGFPKDWVVGEKTGTCANGARND    240
GES-22      MARTVAKVLYGGALTSTSTHTIERWLIGNQTGDATLRAGFPKDWVVGEKTGTCANGARND    240
GES-11      MARTVAKVLYGGALTSTSTHTIERWLIGNQTGDATLRAGFPKDWVVGEKTGTCANGARND    240
GES-12      MARTVAKVLYGGALTSTSTHTIERWLIGNQTGDATLRAGFPKDWVVGEKTGACANGARND    240
            *******:*******************************************:********

GES-35      IGFFKAQERDYAVAVYTTAPKLSAVERDELVASVGQVITQLILSTDK         287
GES-22      IGFFKAQERDYAVAVYTTAPKLSAVERDELVASVGQVITQLILSTDK         287
GES-11      IGFFKAQERDYAVAVYTTAPKLSAVERDELVASVGQVITQLILSTDK         287
GES-12      IGFFKAQERDYAVAVYTTAPKLSAVERDELVASVGQVITQLILSTDK         287
            ***********************************************
```

Nucleotide and amino acid sequence alignments of the *bla*$_{GES-12}$, *bla*$_{GES-22}$ and *bla*$_{GES-35}$ in reference to the *bla*$_{GES-11}$ allele detected in the genomes of IC12s. Nucleotide (A) and amino acid changes (B) were detected in the *bla*$_{GES}$ alleles (highlighted in red). The alignment was done using Clustal Omega (https://www.ebi.ac.uk/Tools/msa/clustalo/).

Supplementary Figure S5.

>*tet(X3)* Ab-IC12-Peru-4
ATGACAATGCGAATAGATACAGACAAACAAATGAATTTACTTAGTGATAAGAACGTTGCAATAATTGGTGGTGGACCCGTTGGACTGACTATGG
CAAAATTATTACAGCAAAACGGCATAGACGTTTCAGTTTACGAAAGAGACAACGACCGAGAGGCAAGAATTTTTGGTGGAACCCTTGACCTACA
CAAAGGTTCAGGTCAGGAAGCAATGAAAAAAGCGGGATTGTTACAAACTTATTATGACTTAGCCTTACCAATGGGTGTAAATATTGCTGATGAA
AAAGGCAATATTTTATCCACAAAAAATGTAAAGCCCGAAAATCGATTTGACAATCCTGAAATAAACAGAAATGACTTAAGGGCTATCTTGTTGA
ATAGTTTAGAAAACGACACGGTTATTTGGGATAGAAAACTTGTTATGCTTGAACCTGGTAAGAAGAAGTGGACACTAACTTTTGAGAATAAACC
GAGTGAAACAGCAGATTTGGTTATTCTTGCCAATGGTGGAATGTCGAAAATAAGGAGCTTTGTTACCGACACGCAAGTTGAAGAAACCGGTACT
TTCAACATCCAAGCTGATATTCTTCAACCGGAAATAAACTGTCCCGGATTTTTTCAGCTATGCAACGGCAACCGATTAATGGCGGGACATCAGG
GCATTTTATTGTTTGCCAATCCCAATAATAATGGTGCATTGTATTTAGGAATTAGTTTTAAAACGCCCGATGAATGGAAAAATAAAATTCCCTT
AGATTTTCAGGACAGAAACAGCGTTGCCGATTTTTTATTGAAAAGATTTTCCAAATGGAGTGAAGTTTACAAACAATTAATACGTTCGGTATCA
ACATTTCAATGCTTGCCCACAAGGAAATTTCCTTTGAACAATGATTGGAAAAGTAAGCGTCCATTACCCATAACGATGATTGGAGATGCTGCTC
ATTTGATGTCGCCTTTTGCAGGACAGGGTGTAAATACGGGATTATTGGATGCTTTGATATTGTCTGAAAACCTTACAAACGGAGAATTTACAAG
TATTGAAAATGCCATCGAAAACTACGAACAACAAATGTTTGTTTATGCAAAAGATACGCAGGACGAATCGACAGAAAACGAAACCGAAATGTTT
AGTCCCAATTTTTCGTTTCAAAAATTATTGAATCTATAA

>*tet(X3)* p34AB
ATGACAATGCGAATAGATACAGACAAACAAATGAATTTACTTAGTGATAAGAACGTTGCAATAATTGGTGGTGGACCCGTTGGACTGACTATGG
CAAAATTATTACAGCAAAACGGCATAGACGTTTCAGTTTACGAAAGAGACAACGACCGAGAGGCAAGAATTTTTGGTGGAACCCTTGACCTACA
CAAAGGTTCAGGTCAGGAAGCAATGAAAAAAGCGGGATTGTTACAAACTTATTATGACTTAGCCTTACCAATGGGTGTAAATATTGCTGATGAA
AAAGGCAATATTTTATCCACAAAAAATGTAAAGCCCGAAAATCGATTTGACAATCCTGAAATAAACAGAAATGACTTAAGGGCTATCTTGTTGA
ATAGTTTAGAAAACGACACGGTTATTTGGGATAGAAAACTTGTTATGCTTGAACCTGGTAAGAAGAAGTGGACACTAACTTTTGAGAATAAACC
GAGTGAAACAGCAGATTTGGTTATTCTTGCCAATGGTGGAATGTCGAAAATAAGGAGCTTTGTTACCGACACGCAAGTTGAAGAAACCGGTACT
TTCAACATCCAAGCTGATATTCTTCAACCGGAAATAAACTGTCCCGGATTTTTTCAGCTATGCAACGGCAACCGATTAATGGCGGGACATCAGG
GCATTTTATTGTTTGCCAATCCCAATAATAATGGTGCATTGTATTTAGGAATTAGTTTTAAAACGCCCGATGAATGGAAAAATAAAATTCCCTT
AGATTTTCAGGACAGAAACAGCGTTGCCGATTTTTTATTGAAAAGATTTTCCAAATGGAGTGAAGTTTACAAACAATTAATACGTTCGGTATCA
ACATTTCAATGCTTGCCCACAAGGAAATTTCCTTTGAACAATGATTGGAAAAGTAACCGTCCATTACCCATAACAATGATTGGCGATGCTGCTC
ATTTGATGTCGCCTTTTGCAGGACAGGGTGTAAATACGGGATTATTGGATGCTTTGATATTGTCTGAAAACCTTACAAACGGAGAATTTACAAG
TATTGAAAATGCCATCGAAAACTACGAACAACAAATGTTTGTTTATGCAAAAGATACGCAGGACGAATCGACAGAAAACGAAACCGAAATGTTT
AGTCCCAATTTTTCGTTTCAAAAATTATTGAATCTATAA

```
IC12-Peru      ATGACAATGCGAATAGATACAGACAAACAAATGAATTTACTTAGTGATAAGAACGTTGCA      60
p34AB          ATGACAATGCGAATAGATACAGACAAACAAATGAATTTACTTAGTGATAAGAACGTTGCA      60
               ************************************************************

IC12-Peru      ATAATTGGTGGTGGACCCGTTGGACTGACTATGGCAAAATTATTACAGCAAAACGGCATA      120
p34AB          ATAATTGGTGGTGGACCCGTTGGACTGACTATGGCAAAATTATTACAGCAAAACGGCATA      120
               ************************************************************

IC12-Peru      GACGTTTCAGTTTACGAAAGAGACAACGACCGAGAGGCAAGAATTTTTGGTGGAACCCTT      180
p34AB          GACGTTTCAGTTTACGAAAGAGACAACGACCGAGAGGCAAGAATTTTTGGTGGAACCCTT      180
               ************************************************************

IC12-Peru      GACCTACACAAAGGTTCAGGTCAGGAAGCAATGAAAAAAGCGGGATTGTTACAAACTTAT      240
p34AB          GACCTACACAAAGGTTCAGGTCAGGAAGCAATGAAAAAAGCGGGATTGTTACAAACTTAT      240
               ************************************************************

IC12-Peru      TATGACTTAGCCTTACCAATGGGTGTAAATATTGCTGATGAAAAAGGCAATATTTTATCC      300
p34AB          TATGACTTAGCCTTACCAATGGGTGTAAATATTGCTGATGAAAAAGGCAATATTTTATCC      300
               ************************************************************

IC12-Peru      ACAAAAAATGTAAAGCCCGAAAATCGATTTGACAATCCTGAAATAAACAGAAATGACTTA      360
p34AB          ACAAAAAATGTAAAGCCCGAAAATCGATTTGACAATCCTGAAATAAACAGAAATGACTTA      360
               ************************************************************

IC12-Peru      AGGGCTATCTTGTTGAATAGTTTAGAAAACGACACGGTTATTTGGGATAGAAAACTTGTT      420
p34AB          AGGGCTATCTTGTTGAATAGTTTAGAAAACGACACGGTTATTTGGGATAGAAAACTTGTT      420
               ************************************************************

IC12-Peru      ATGCTTGAACCTGGTAAGAAGAAGTGGACACTAACTTTTGAGAATAAACCGAGTGAAACA      480
p34AB          ATGCTTGAACCTGGTAAGAAGAAGTGGACACTAACTTTTGAGAATAAACCGAGTGAAACA      480
               ************************************************************

IC12-Peru      GCAGATTTGGTTATTCTTGCCAATGGTGGAATGTCGAAAATAAGGAGCTTTGTTACCGAC      540
p34AB          GCAGATTTGGTTATTCTTGCCAATGGTGGAATGTCGAAAATAAGGAGCTTTGTTACCGAC      540
               ************************************************************

IC12-Peru      ACGCAAGTTGAAGAAACCGGTACTTTCAACATCCAAGCTGATATTCTTCAACCGGAAATA      600
p34AB          ACGCAAGTTGAAGAAACCGGTACTTTCAACATCCAAGCTGATATTCTTCAACCGGAAATA      600
               ************************************************************

IC12-Peru      AACTGTCCCGGATTTTTTCAGCTATGCAACGGCAACCGATTAATGGCGGGACATCAGGGC      660
p34AB          AACTGTCCCGGATTTTTTCAGCTATGCAACGGCAACCGATTAATGGCGGGACATCAGGGC      660
               ************************************************************
```

```
IC12-Peru      ATTTTATTGTTTGCCAATCCCAATAATAATGGTGCATTGTATTTAGGAATTAGTTTTAAA      720
p34AB          ATTTTATTGTTTGCCAATCCCAATAATAATGGTGCATTGTATTTAGGAATTAGTTTTAAA      720
               ************************************************************

IC12-Peru      ACGCCCGATGAATGGAAAAATAAAATTCCCTTAGATTTTCAGGACAGAAACAGCGTTGCC      780
p34AB          ACGCCCGATGAATGGAAAAATAAAATTCCCTTAGATTTTCAGGACAGAAACAGCGTTGCC      780
               ************************************************************

IC12-Peru      GATTTTTTATTGAAAAGATTTTCCAAATGGAGTGAAGTTTACAAACAATTAATACGTTCG      840
p34AB          GATTTTTTATTGAAAAGATTTTCCAAATGGAGTGAAGTTTACAAACAATTAATACGTTCG      840
               ************************************************************

IC12-Peru      GTATCAACATTTCAATGCTTGCCCACAAGGAAATTTCCTTTGAACAATGATTGGAAAAGT      900
p34AB          GTATCAACATTTCAATGCTTGCCCACAAGGAAATTTCCTTTGAACAATGATTGGAAAAGT      900
               ************************************************************

IC12-Peru      AAGCGTCCATTACCCATAACGATGATTGGAGATGCTGCTCATTTGATGTCGCCTTTTGCA      960
p34AB          AACCGTCCATTACCCATAACAATGATTGGCGATGCTGCTCATTTGATGTCGCCTTTTGCA      960
               ** ***************** ******** ******************************

IC12-Peru      GGACAGGGTGTAAATACGGGATTATTGGATGCTTTGATATTGTCTGAAAACCTTACAAAC      1020
p34AB          GGACAGGGTGTAAATACGGGATTATTGGATGCTTTGATATTGTCTGAAAACCTTACAAAC      1020
               ************************************************************

IC12-Peru      GGAGAATTTACAAGTATTGAAAATGCCATCGAAAACTACGAACAACAAATGTTTGTTTAT      1080
p34AB          GGAGAATTTACAAGTATTGAAAATGCCATCGAAAACTACGAACAACAAATGTTTGTTTAT      1080
               ************************************************************

IC12-Peru      GCAAAAGATACGCAGGACGAATCGACAGAAAACGAAACCGAAATGTTTAGTCCCAATTTT      1140
p34AB          GCAAAAGATACGCAGGACGAATCGACAGAAAACGAAACCGAAATGTTTAGTCCCAATTTT      1140
               ************************************************************

IC12-Peru      TCGTTTCAAAAATTATTGAATCTATAA   1167
p34AB          TCGTTTCAAAAATTATTGAATCTATAA   1167
               ***************************
```

Nucleotide sequence alignment of the *tet*(X3) allele that was detected in the genomes of 3 isolates from International Clone 12 (represented by Ab-IC12-Peru-4) and the first described *tet*(X3) allele in p34AB (GenBank: MK134375.1). Three single nucleotide changes were detected (highlighted in yellow). The alignment was done using Clustal Omega (https://www.ebi.ac.uk/Tools/msa/clustalo/).

Supplementary Figure S6.

>aadB Ab-IC12-Lebanon-1
ATGGACACAACGCAGGTCACATTGATACACAAAATTCTAGCTGCGGCAGATGAGCGAAATCTGCCGCTCTGGATCGGTGGGGGCTGGGCGATCG
ATGCACGGCTAGGGCGTGTAACACGCAAGCACGATGATATTGATCTGACGTTTCCCGGCGAGAGGCGCGGCGAGCTCGAGGCAATAGTTGAAAT
GCTCGGCGGGCGCGTCATGGAGGAGTTGGACTATGGATTCTTAGCGGAGATCGGGGATGAGTTACTTGACTGCGAACCTGCTTGGTGGGCAGAC
GAAGCGTATGAAATCGCGGAGGCTCCGCAGGGCTCGTGCCCAGAGGCGGCTGAGGGCGTCATCGCCGGGCGGCCAGTCCGTTGTAACAGCTGGG
AGGCGATCATCTGGGATTACTTTTACTATGCCGATGAAGTACCACCAGTGGACTGGCCTACAAAGCACATAGAGTCCTACAGGCTCGCATGCAC
CTCACTCGGGGCGGAAAAGGTTGAGGTCTTGCGTGCCGCTTTCAGGTCGCGATATGCGGCCTAA

>aadB Ab-IC12-Egypt-1
ATGGACACAACGCAGGTCGCATTGATACACCAAATTCTAGCTGCGGCAGATGAGCGAAATCTGCCGCTCTGGATCGGTGGGGGCTGGGCGATCG
ATGCACGGCTAGGGCGTGTAACACGCAAGCACGATGATATTGATCTGACTTTTCCCGGCGAGAGGCGCGGCGAGCTCGAGGCAATAGTTGAAAT
GCTCGGCGGGCGCGTCACGGAGGAGTTGGACTATGGATTCTTAGCGGAGATCGGGGATGAGTTACTTGACTGCGAACCTGCTTGGTGGGCAGAC
GAAGCGTATGAAATCGCGGAGGCTCCGCAGGGCTCGTGCCCAGAGGCGGCTGAGGGTGTCATCGCCGGGCGGCCAGTCCGTTGTAACAGCTGGG
AGGCGATCATCTGGGATTACTTTTACTATGCCGATGAAGTACCACCAGTGGACTGGCCTACAAAGCACATAGAGTCCTACAGGTTCGCATGCAC
CTCACTCGGGGCGGAAAAGGTTGAGGTCTTGCGTGCCGCTTTCAGGTCGCGATATGCGGCCTAA

```
aadB Ab-IC12-Lebanon-1      ATGGACACAACGCAGGTCACATTGATACACAAAATTCTAGCTGCGGCAGATGAGCGAAAT   60
aadB Ab-IC12-Egypt-1        ATGGACACAACGCAGGTCGCATTGATACACCAAATTCTAGCTGCGGCAGATGAGCGAAAT   60
                            ***************** *********** ******************************

aadB Ab-IC12-Lebanon-1      CTGCCGCTCTGGATCGGTGGGGGCTGGGCGATCGATGCACGGCTAGGGCGTGTAACACGC   120
aadB Ab-IC12-Egypt-1        CTGCCGCTCTGGATCGGTGGGGGCTGGGCGATCGATGCACGGCTAGGGCGTGTAACACGC   120
                            ************************************************************

aadB Ab-IC12-Lebanon-1      AAGCACGATGATATTGATCTGACGTTTCCCGGCGAGAGGCGCGGCGAGCTCGAGGCAATA   180
aadB Ab-IC12-Egypt-1        AAGCACGATGATATTGATCTGACTTTTCCCGGCGAGAGGCGCGGCGAGCTCGAGGCAATA   180
                            *********************** ************************************

aadB Ab-IC12-Lebanon-1      GTTGAAATGCTCGGCGGGCGCGTCATGGAGGAGTTGGACTATGGATTCTTAGCGGAGATC   240
aadB Ab-IC12-Egypt-1        GTTGAAATGCTCGGCGGGCGCGTCACGGAGGAGTTGGACTATGGATTCTTAGCGGAGATC   240
                            ************************* **********************************

aadB Ab-IC12-Lebanon-1      GGGGATGAGTTACTTGACTGCGAACCTGCTTGGTGGGCAGACGAAGCGTATGAAATCGCG   300
aadB Ab-IC12-Egypt-1        GGGGATGAGTTACTTGACTGCGAACCTGCTTGGTGGGCAGACGAAGCGTATGAAATCGCG   300
                            ************************************************************

aadB Ab-IC12-Lebanon-1      GAGGCTCCGCAGGGCTCGTGCCCAGAGGCGGCTGAGGGCGTCATCGCCGGGCGGCCAGTC   360
aadB Ab-IC12-Egypt-1        GAGGCTCCGCAGGGCTCGTGCCCAGAGGCGGCTGAGGGTGTCATCGCCGGGCGGCCAGTC   360
                            ************************************** *********************

aadB Ab-IC12-Lebanon-1      CGTTGTAACAGCTGGGAGGCGATCATCTGGGATTACTTTTACTATGCCGATGAAGTACCA   420
aadB Ab-IC12-Egypt-1        CGTTGTAACAGCTGGGAGGCGATCATCTGGGATTACTTTTACTATGCCGATGAAGTACCA   420
                            ************************************************************

aadB Ab-IC12-Lebanon-1      CCAGTGGACTGGCCTACAAAGCACATAGAGTCCTACAGGCTCGCATGCACCTCACTCGGG   480
aadB Ab-IC12-Egypt-1        CCAGTGGACTGGCCTACAAAGCACATAGAGTCCTACAGGTTCGCATGCACCTCACTCGGG   480
                            *************************************** ********************

aadB Ab-IC12-Lebanon-1      GCGGAAAAGGTTGAGGTCTTGCGTGCCGCTTTCAGGTCGCGATATGCGGCCTAA         534
aadB Ab-IC12-Egypt-1        GCGGAAAAGGTTGAGGTCTTGCGTGCCGCTTTCAGGTCGCGATATGCGGCCTAA         534
                            ******************************************************
```

Nucleotide sequence alignment of two different *aadB* alleles detected in the genomes of 5 isolates (represented by *aadB*$_{\text{Ab-IC12-Lebanon-1}}$) and 6 isolates (represented by *aadB*$_{\text{Ab-IC12-Egypte-1}}$). Six single-nucleotide changes were detected (highlighted in yellow). The alignment was done using Clustal Omega (https://www.ebi.ac.uk/Tools/msa/clustalo/).

Supplementary Figure S7.

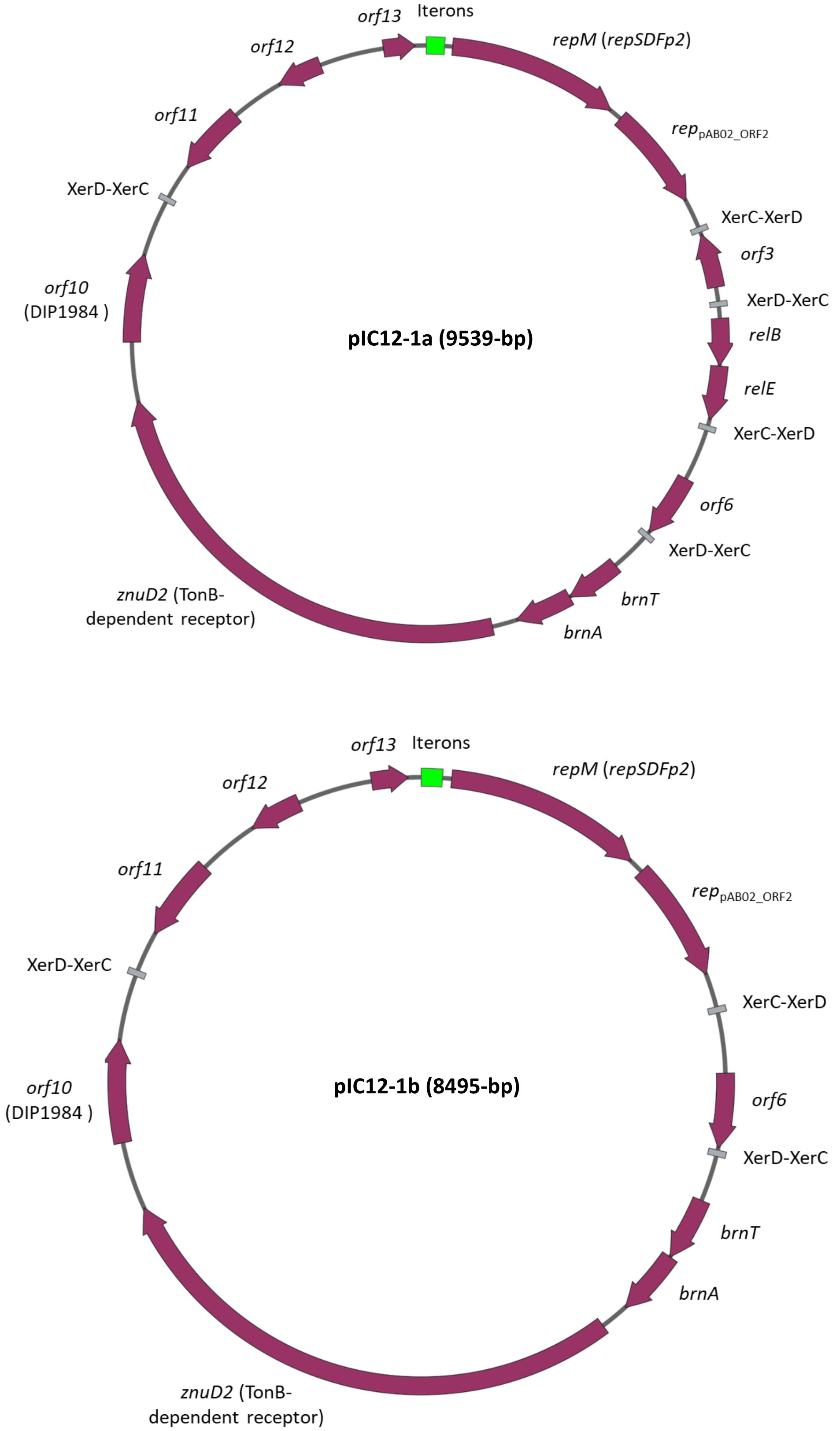

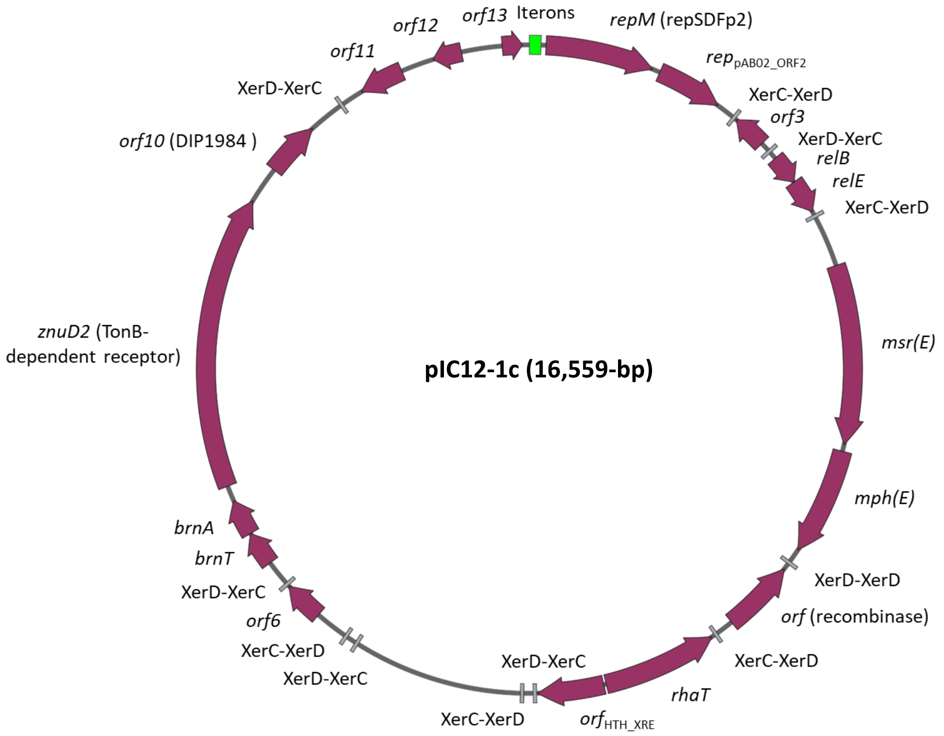
pIC12-1c (16,559-bp)
orf13
Iterons
repM (repSDFp2)
rep_{pAB02_ORF2}
XerC-XerD
orf3
XerD-XerC
relB
relE
XerC-XerD
msr(E)
mph(E)
XerD-XerD
orf (recombinase)
XerC-XerD
rhaT
orf_{HTH_XRE}
XerD-XerC
XerC-XerD
XerD-XerC
XerC-XerD
orf6
XerD-XerC
brnT
brnA
znuD2 (TonB-dependent receptor)
orf10 (DIP1984)
XerD-XerC
orf11
orf12

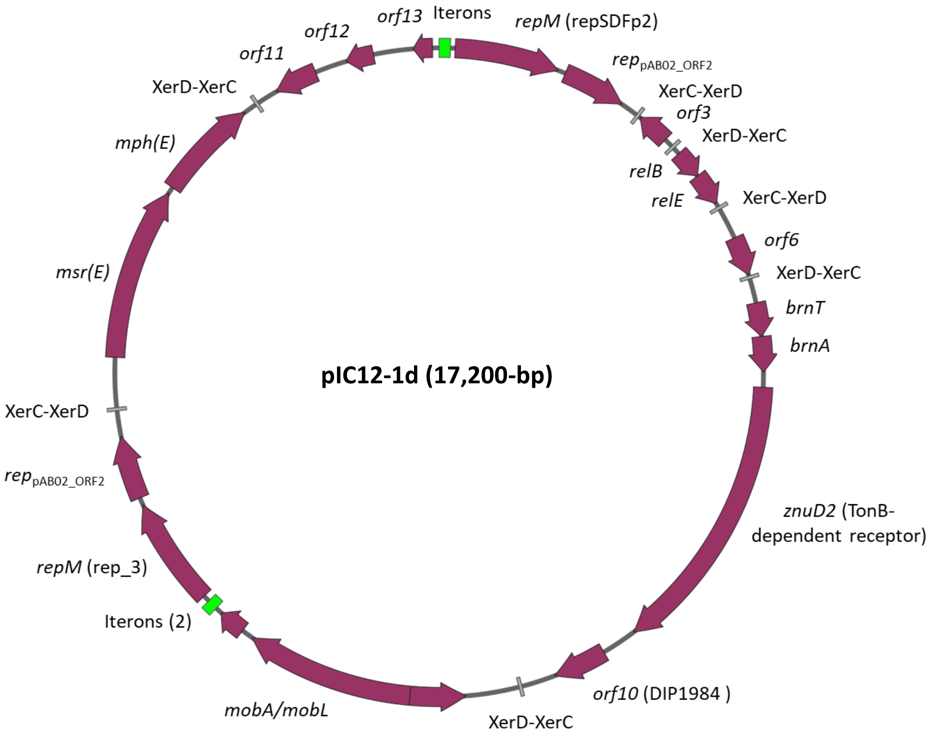
pIC12-1d (17,200-bp)
orf13
Iterons
repM (repSDFp2)
rep_{pAB02_ORF2}
XerC-XerD
orf3
XerD-XerC
relB
relE
XerC-XerD
orf6
XerD-XerC
brnT
brnA
znuD2 (TonB-dependent receptor)
orf10 (DIP1984)
XerD-XerC
mobA/mobL
Iterons (2)
repM (rep_3)
rep_{pAB02_ORF2}
XerC-XerD
msr(E)
mph(E)
XerD-XerC
orf11
orf12

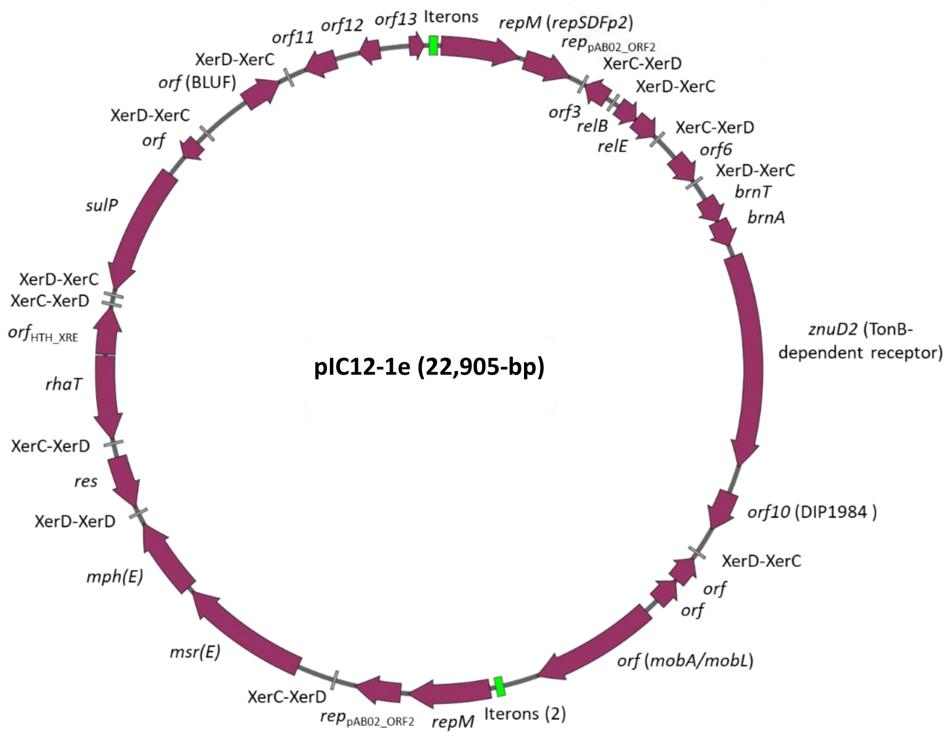
orf13
Iterons
repM (repSDFp2)
rep pAB02_ORF2
XerC-XerD
XerD-XerC
orf3
relB
relE
XerC-XerD
orf6
XerD-XerC
brnT
brnA
znuD2 (TonB-dependent receptor)
orf10 (DIP1984)
XerD-XerC
orf
orf
orf (mobA/mobL)
Iterons (2)
repM
rep pAB02_ORF2
XerC-XerD
msr(E)
mph(E)
XerD-XerD
res
XerC-XerD
rhaT
orf HTH_XRE
XerC-XerD
XerD-XerC
sulP
orf
XerD-XerC
orf (BLUF)
XerD-XerC
orf11
orf12
pIC12-1e (22,905-bp)

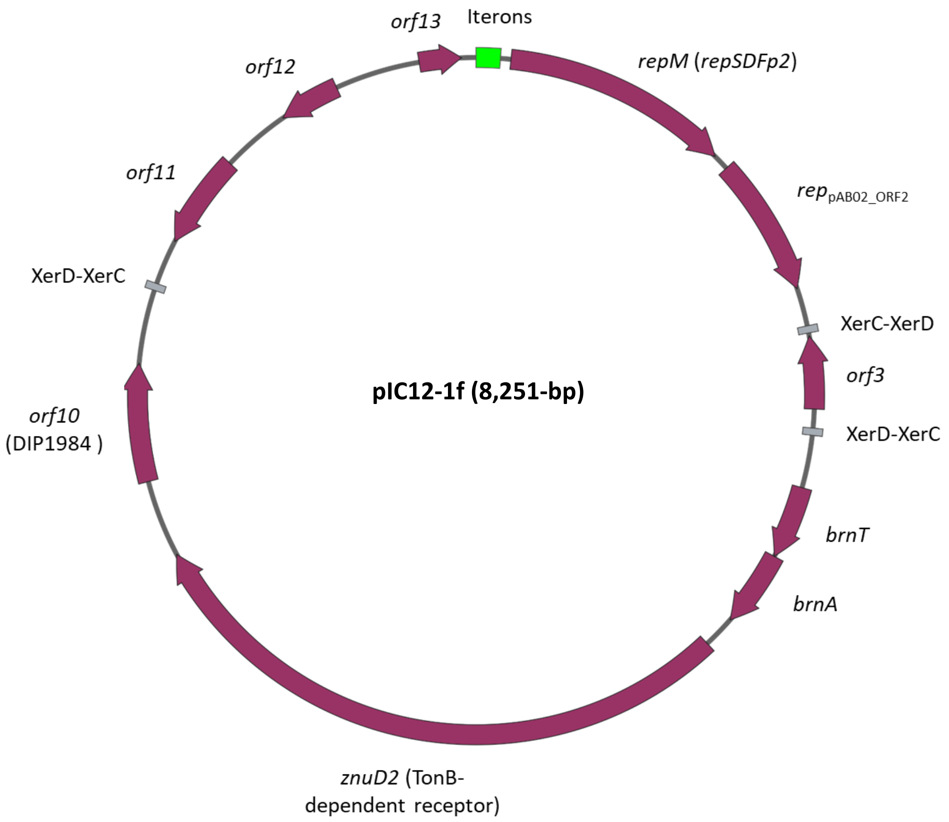
orf13
Iterons
repM (repSDFp2)
orf12
orf11
rep pAB02_ORF2
XerD-XerC
XerC-XerD
orf3
XerD-XerC
orf10
(DIP1984)
brnT
brnA
znuD2 (TonB-dependent receptor)
pIC12-1f (8,251-bp)

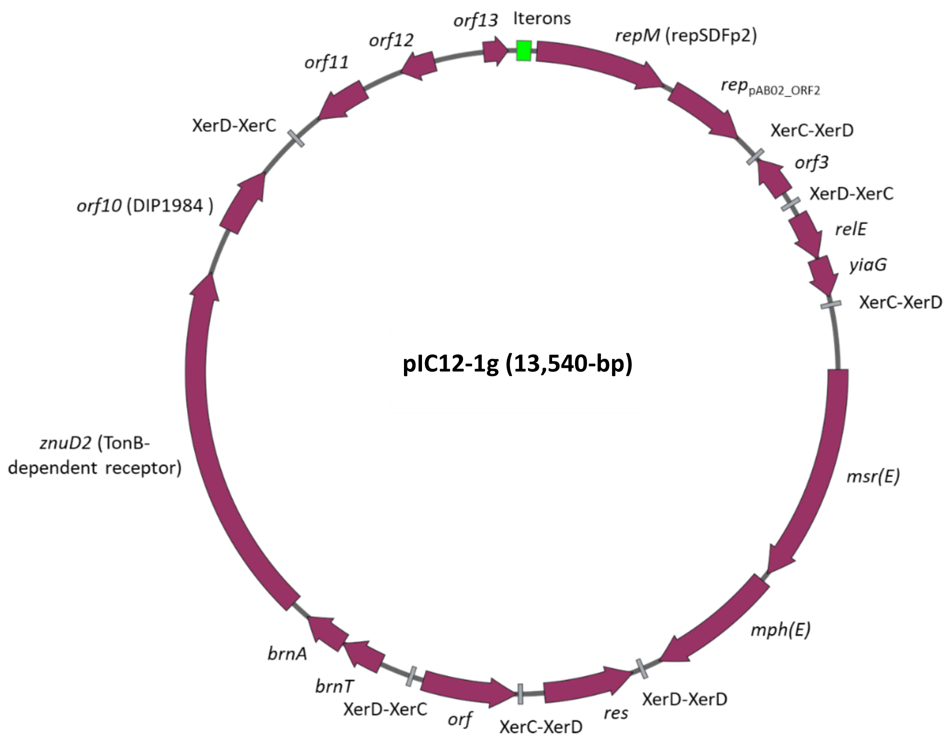

Graphic representation of seven versions of pIC12-1 (a, b, c, d, e, f, and g). Genes and open reading frames (*orf*) were shown as plum arrow shapes, with the arrowhead indicating the direction of transcription.

Supplementary Figure S8.

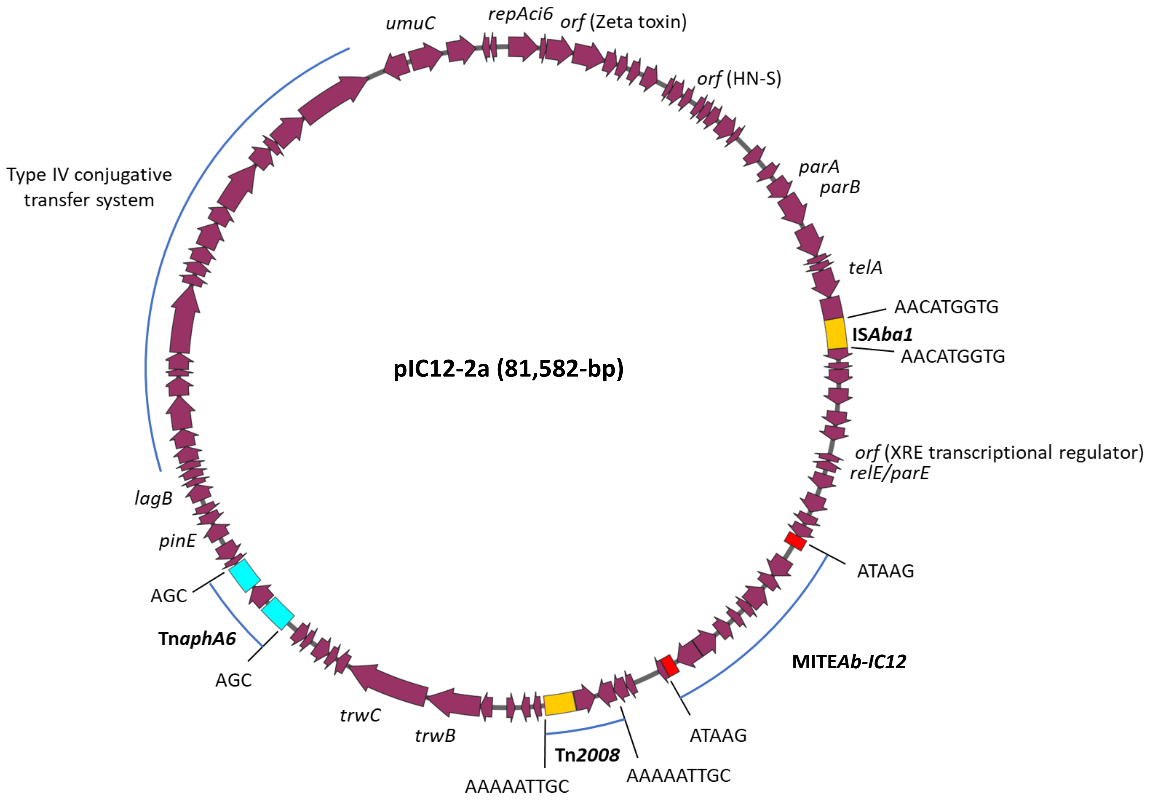

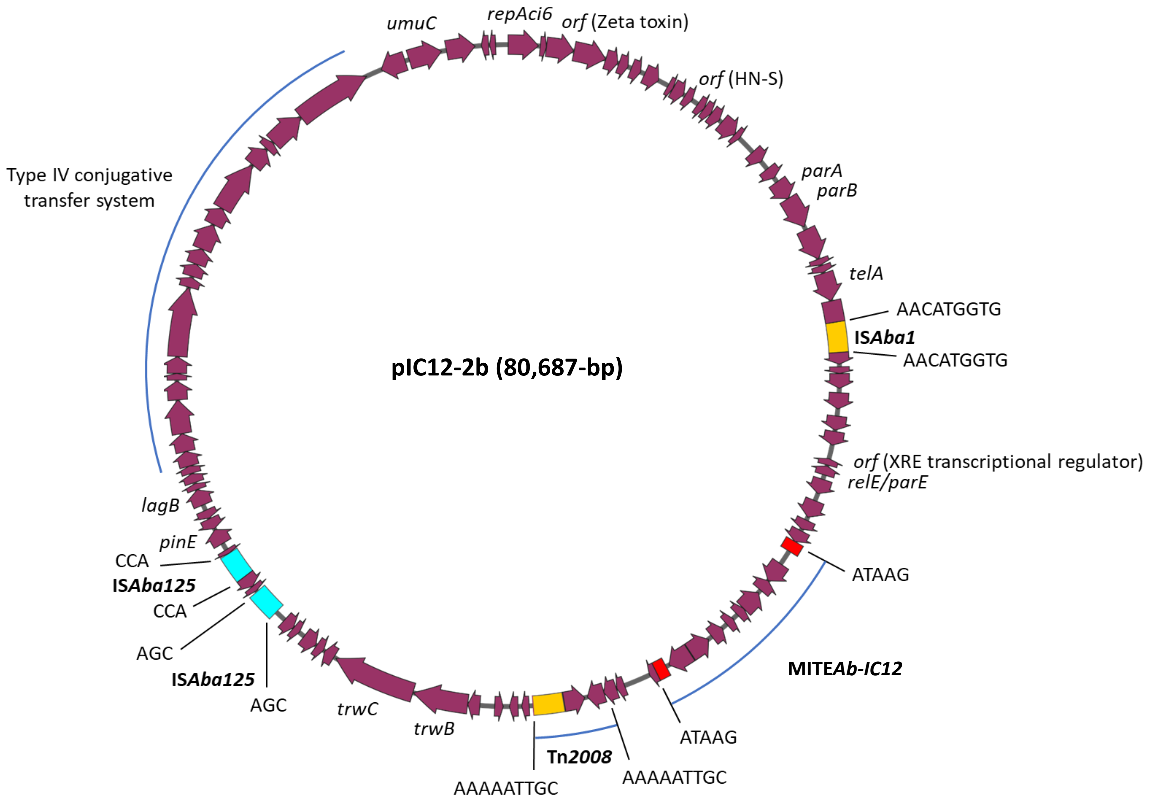

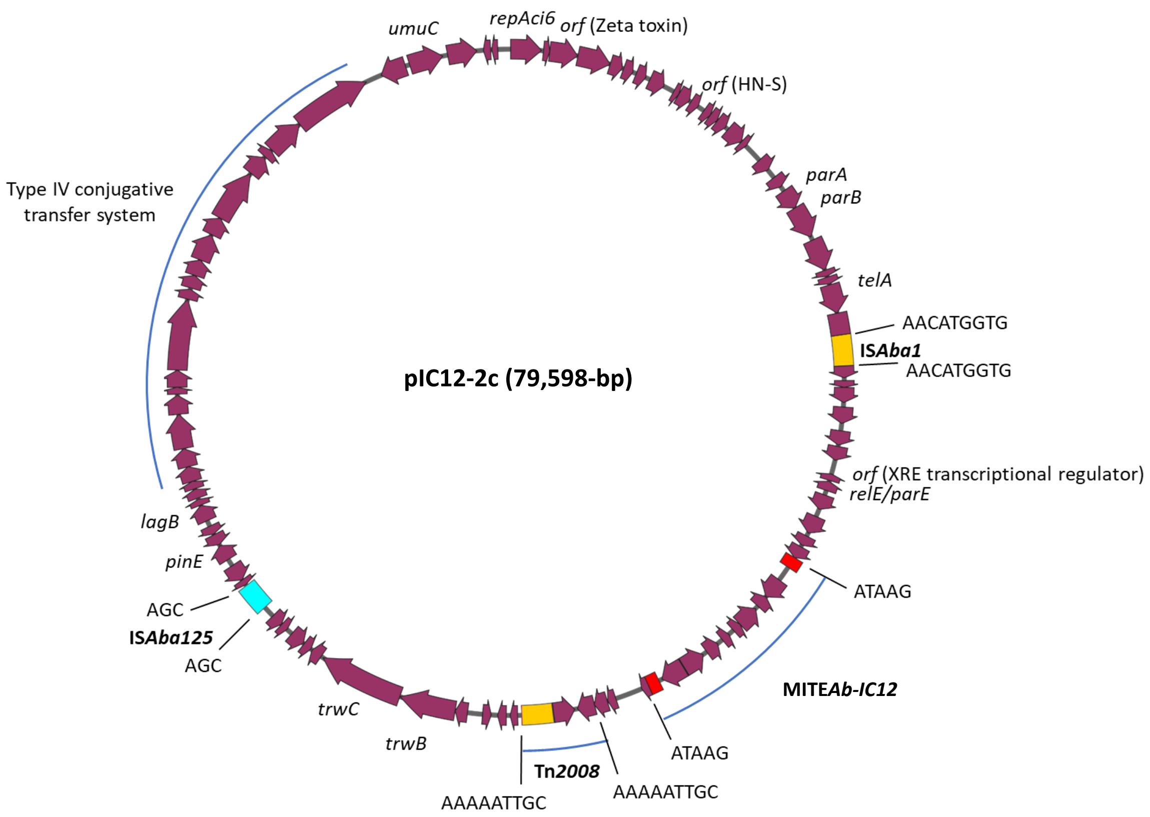
umuC
repAci6
orf (Zeta toxin)
orf (HN-S)
parA
parB
telA
AACATGGTG
ISAba1
AACATGGTG
pIC12-2c (79,598-bp)
orf (XRE transcriptional regulator)
relE/parE
ATAAG
MITEAb-IC12
ATAAG
AAAAATTGC
Tn2008
AAAAATTGC
trwB
trwC
AGC
ISAba125
AGC
pinE
lagB
Type IV conjugative transfer system

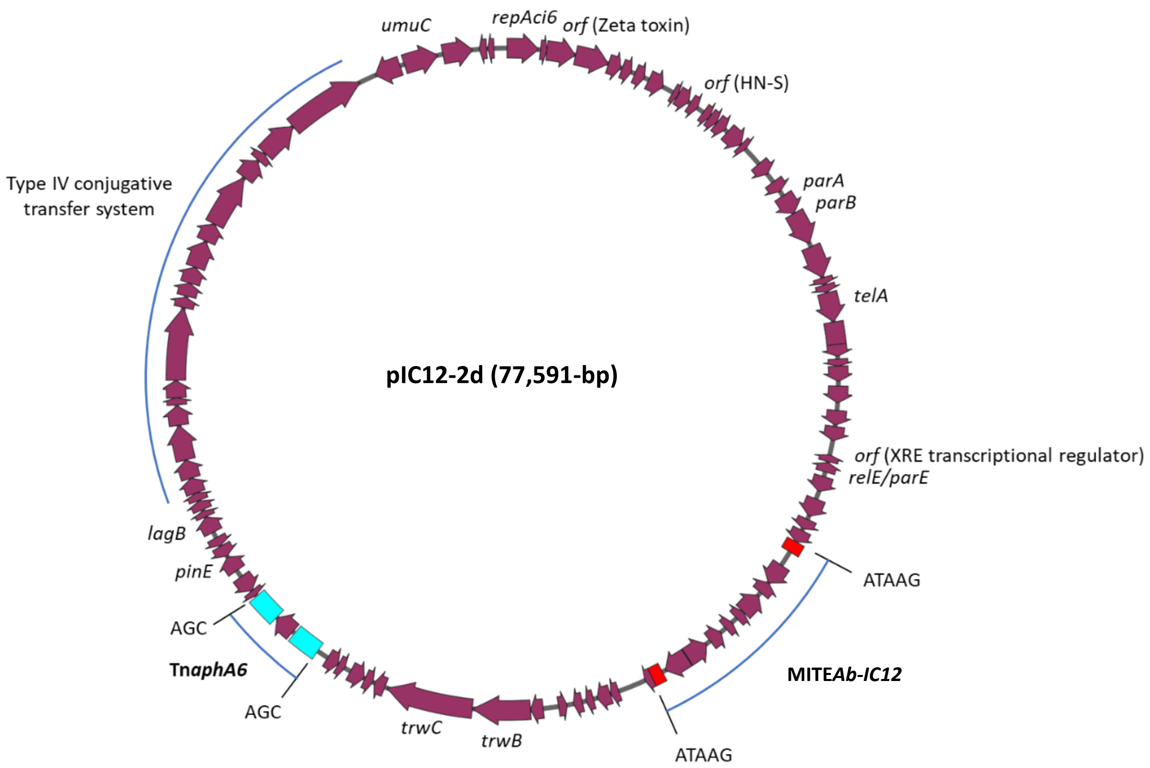
umuC
repAci6
orf (Zeta toxin)
orf (HN-S)
parA
parB
telA
pIC12-2d (77,591-bp)
orf (XRE transcriptional regulator)
relE/parE
ATAAG
MITEAb-IC12
ATAAG
trwB
trwC
AGC
TnaphA6
AGC
pinE
lagB
Type IV conjugative transfer system

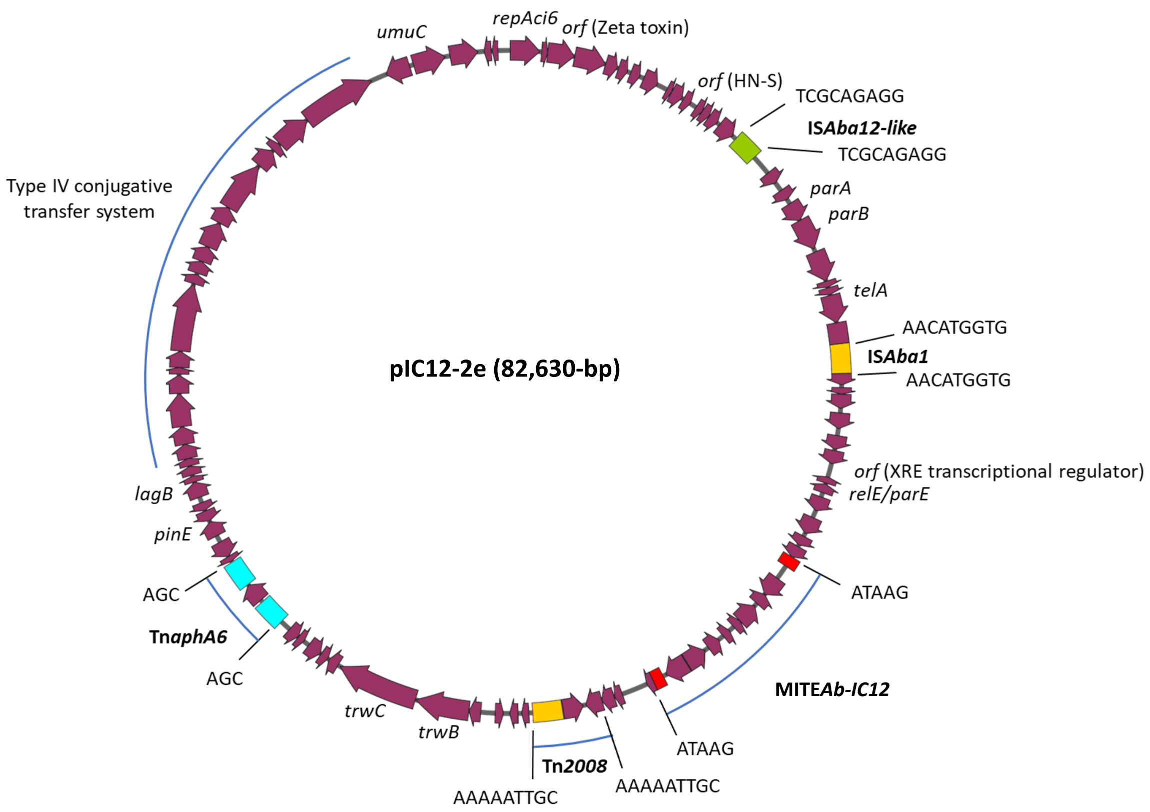
pIC12-2e (82,630-bp)
umuC
repAci6
orf (Zeta toxin)
orf (HN-S)
TCGCAGAGG
ISAba12-like
TCGCAGAGG
parA
parB
telA
AACATGGTG
ISAba1
AACATGGTG
orf (XRE transcriptional regulator)
relE/parE
ATAAG
MITEAb-IC12
ATAAG
Tn2008
AAAAATTGC
AAAAATTGC
trwB
trwC
AGC
TnaphA6
AGC
pinE
lagB
Type IV conjugative transfer system

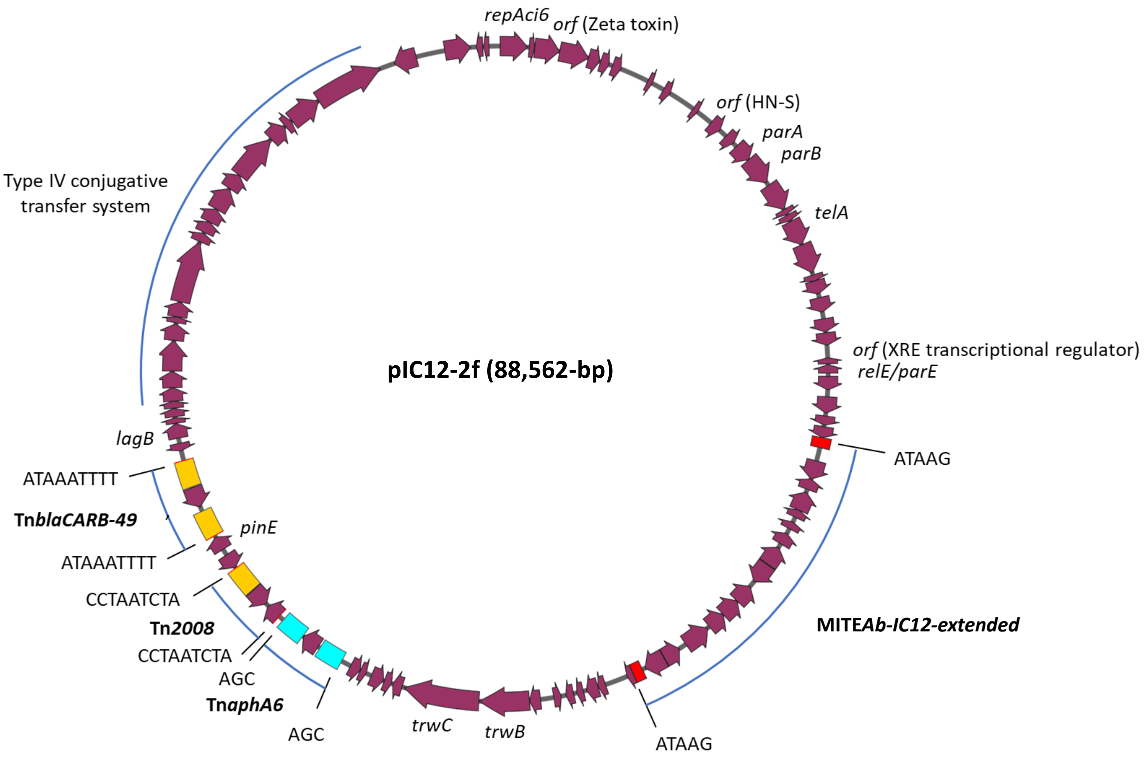
pIC12-2f (88,562-bp)
repAci6
orf (Zeta toxin)
orf (HN-S)
parA
parB
telA
orf (XRE transcriptional regulator)
relE/parE
ATAAG
MITEAb-IC12-extended
ATAAG
trwB
trwC
AGC
TnaphA6
AGC
CCTAATCTA
Tn2008
CCTAATCTA
pinE
ATAAATTTT
TnblaCARB-49
ATAAATTTT
lagB
Type IV conjugative transfer system

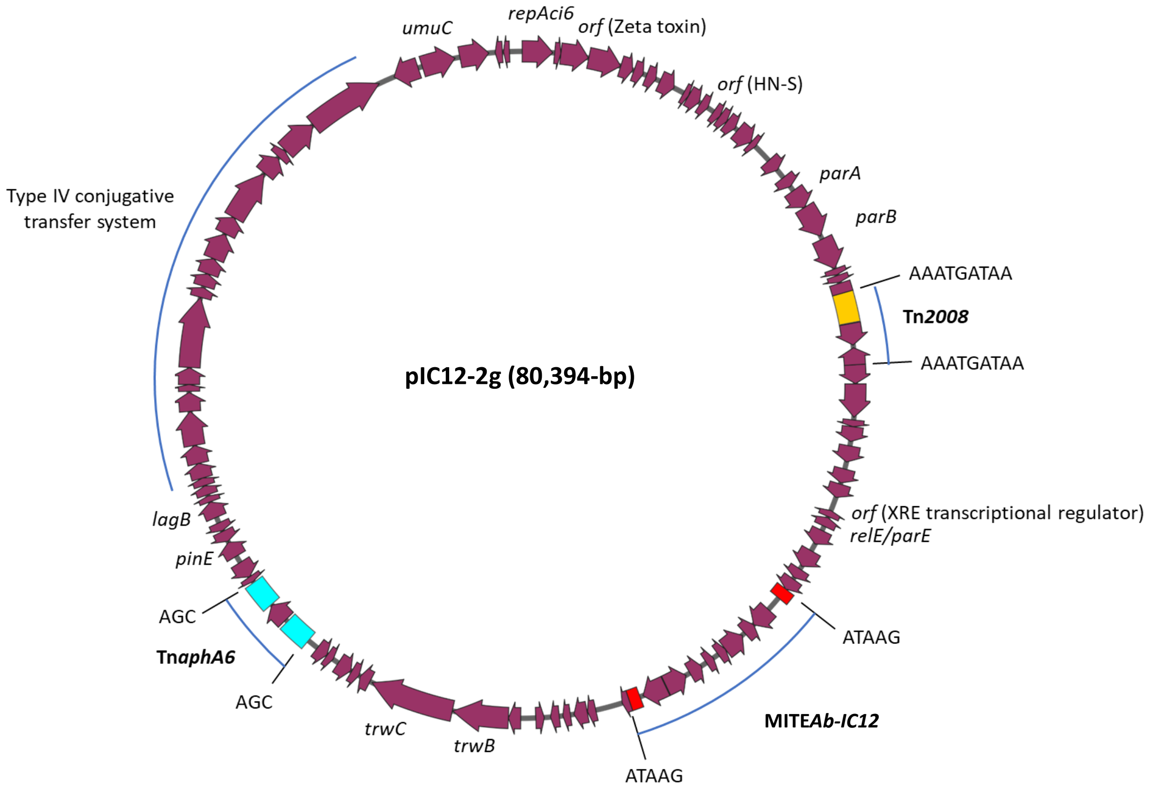

Graphic representation of seven versions of pIC12-2 (a, b, c, d, e, f, and g). Genes and open reading frames (*orf*) were shown as plum arrow shapes, with the arrowhead indicating the direction of transcription.

Supplementary Figure S9.

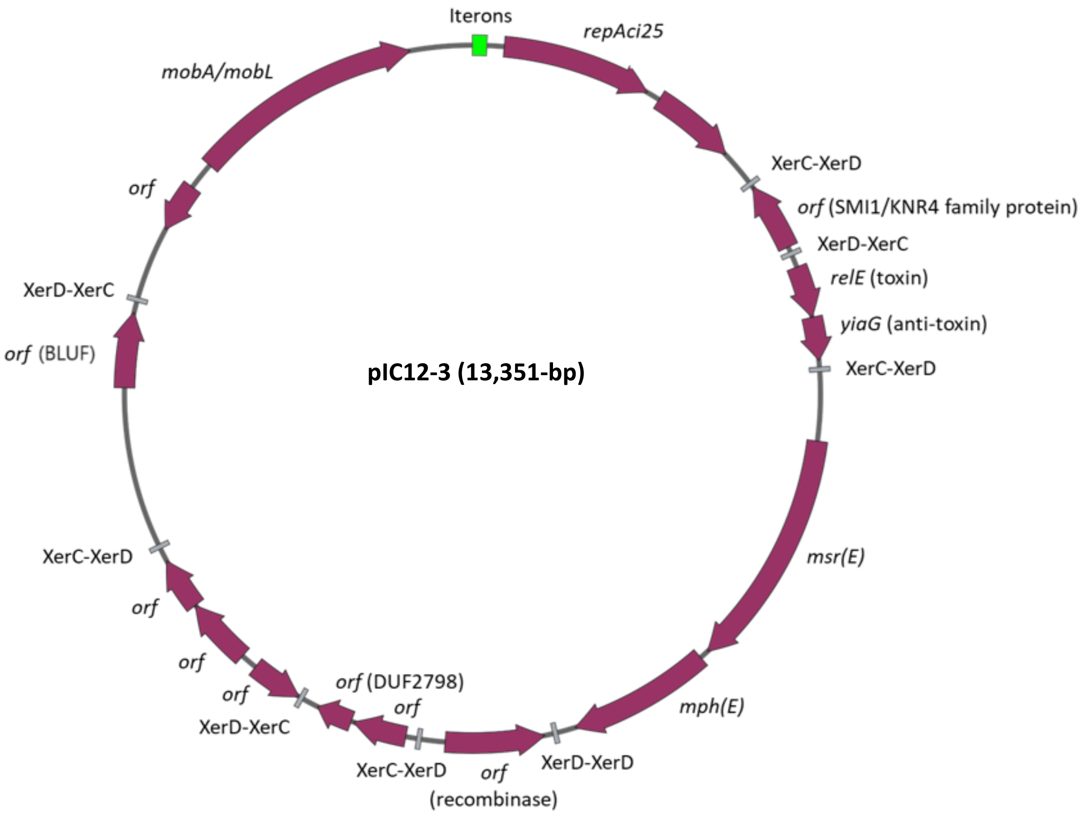

Graphic representation of pIC12-3. Genes and open reading frames (*orf*) were shown as plum arrow shapes, with the arrowhead indicating the direction of transcription.

Supplementary Figure S10.

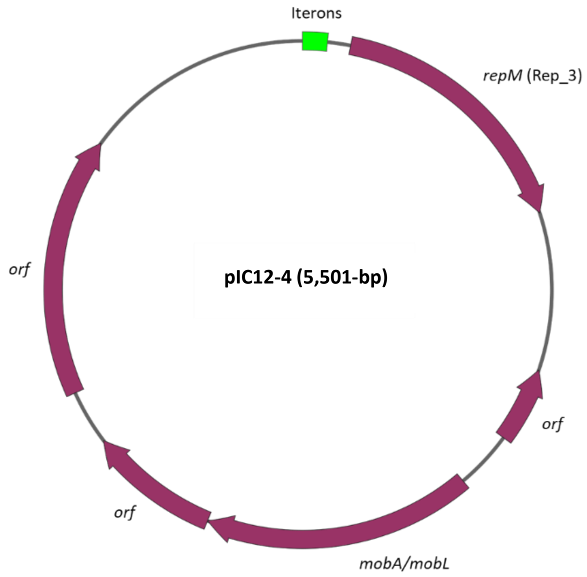

Graphic representation of pIC12-4. Genes and open reading frames (*orf*) were shown as plum arrow shapes, with the arrowhead indicating the direction of transcription.

Supplementary Figure S11.

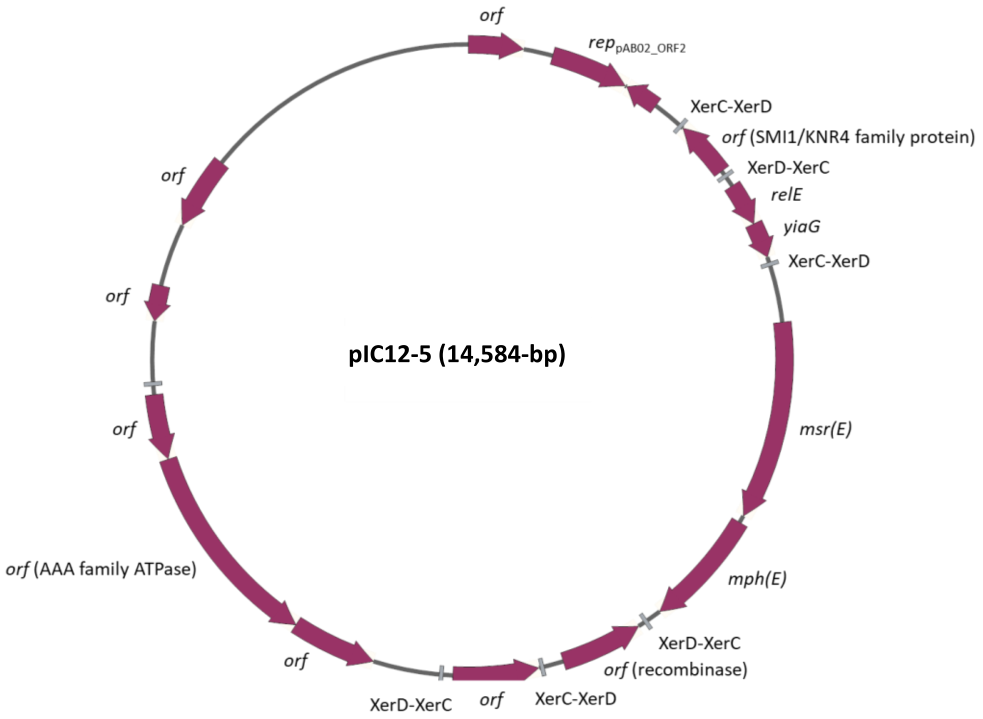

Graphic representation of pIC12-5. Genes and open reading frames (*orf*) were shown as plum arrow shapes, with the arrowhead indicating the direction of transcription.

Supplementary Figure S12.

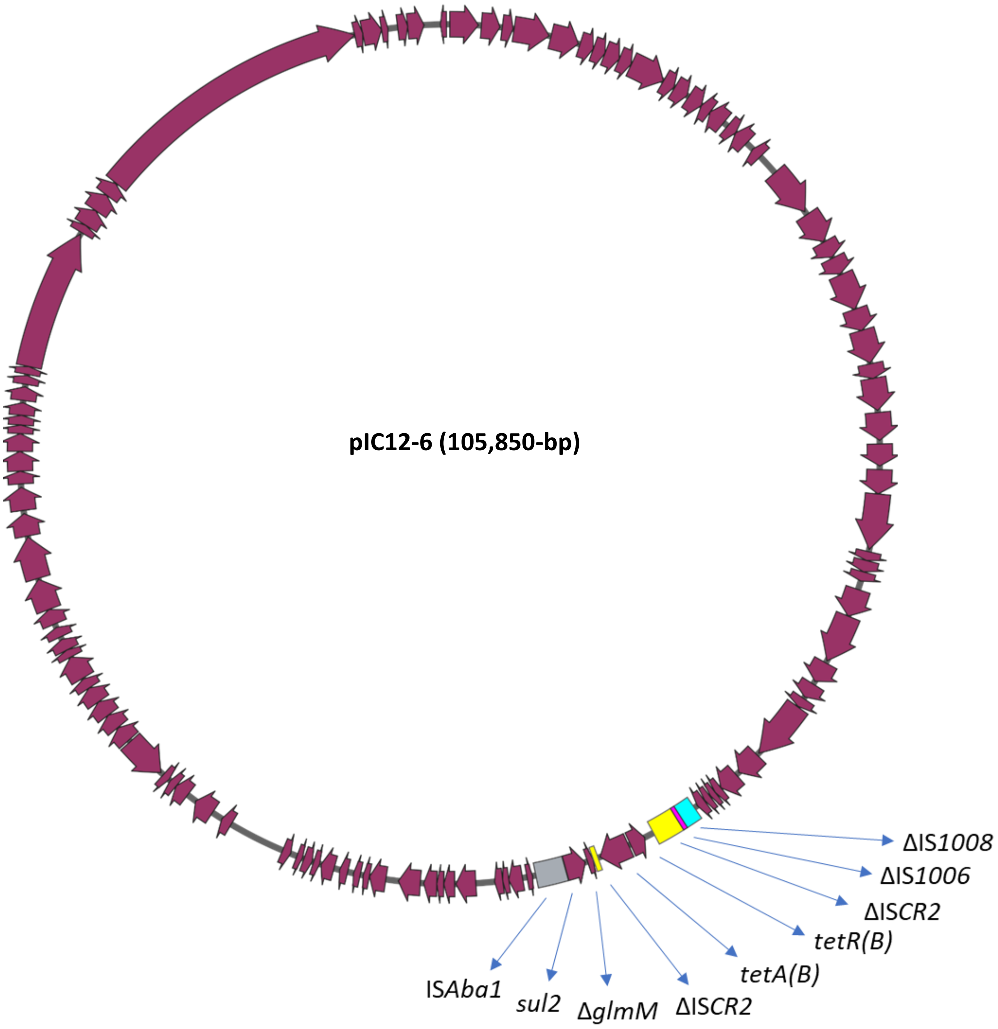

Graphic representation of pIC12-6. Genes and open reading frames (*orf*) were shown as plum arrow shapes, with the arrowhead indicating the direction of transcription.

Supplementary Figure S13.

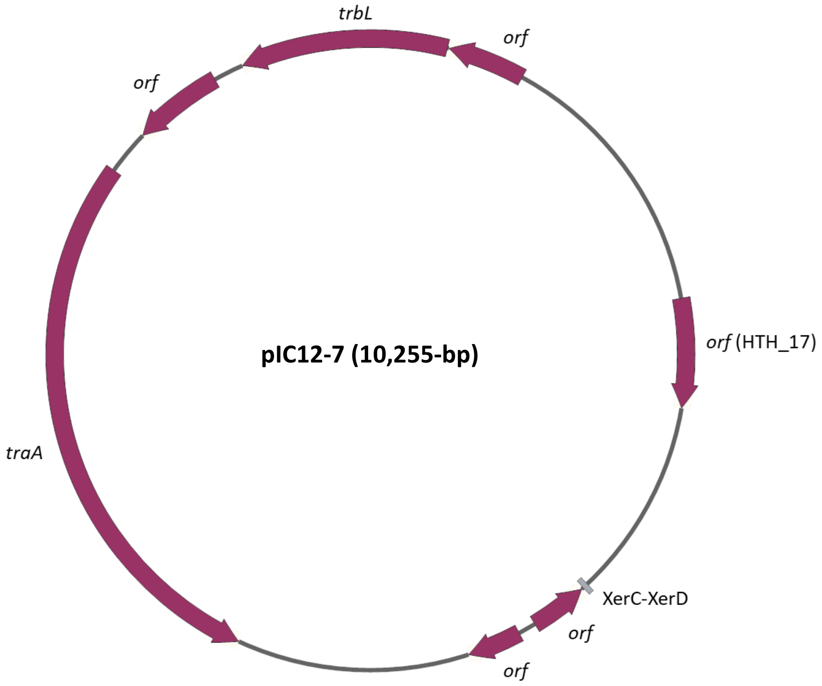

Graphic representation of pIC12-7. Genes and open reading frames (*orf*) were shown as plum arrow shapes, with the arrowhead indicating the direction of transcription.

Supplementary Figure S14.

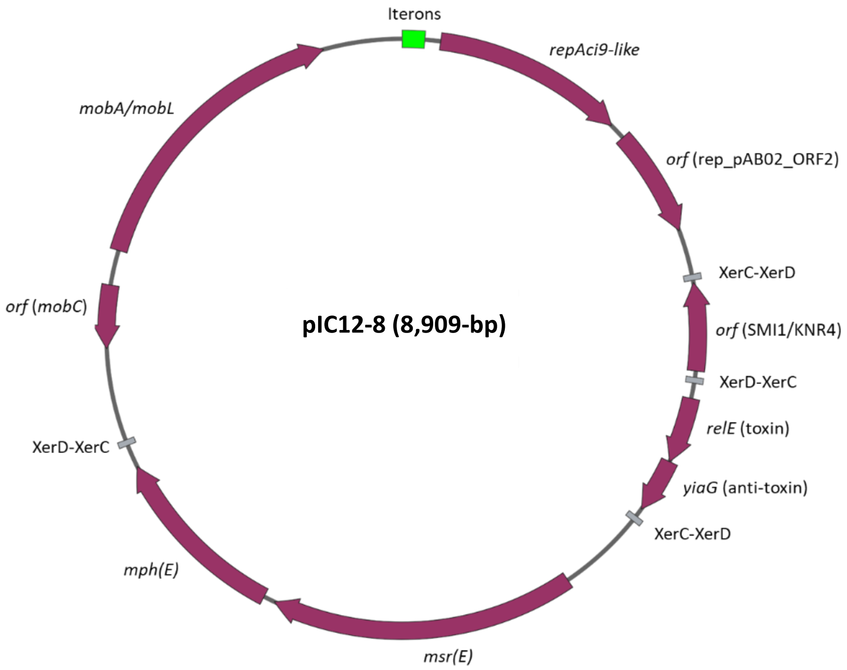

Graphic representation of pIC12-8. Genes and open reading frames (*orf*) were shown as plum arrow shapes, with the arrowhead indicating the direction of transcription.

Supplementary Figure S15.

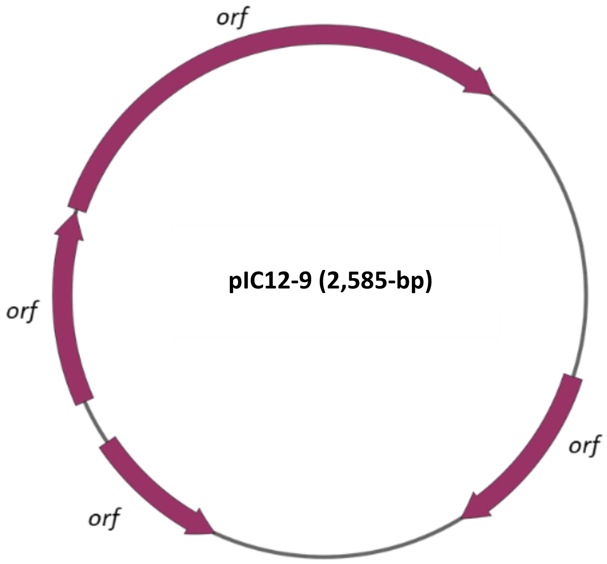

Graphic representation of pIC12-9. Genes and open reading frames (*orf*) were shown as plum arrow shapes, with the arrowhead indicating the direction of transcription.
